# Regulation of low-density lipoprotein receptor expression in triple negative breast cancer by EGFR-MAPK signaling

**DOI:** 10.1038/s41598-021-97327-y

**Published:** 2021-09-09

**Authors:** Tiffany Scully, Nathan Kase, Emily J. Gallagher, Derek LeRoith

**Affiliations:** 1 Division of Endocrinology, Diabetes and Bone Disease, Department of Medicine, Icahn School of Medicine at Mount Sinai, One Gustave L. Levy Place, Box 1055, New York, NY 10029 USA; 2 Tisch Cancer Institute at Mount Sinai, Icahn School of Medicine at Mount Sinai, One Gustave L. Levy Place, New York, NY 10029 USA

**Keywords:** Breast cancer, Growth factor signalling

## Abstract

Expression of the low-density lipoprotein receptor (LDLR) has been shown to play a critical role in hypercholesterolemia-associated breast cancer growth and is associated with shorter recurrence-free survival in human breast cancer studies. We sought to identify how circulating LDL cholesterol and tumor LDLR might accelerate oncogenic processes by determining whether increased LDLR expression and cholesterol uptake are associated with the activation of the epidermal growth factor receptor (EGFR) signaling pathway in triple negative breast cancer (TNBC) cell lines. EGF stimulation of MDA-MB-468 (MDA468) cells activated p44/42MAPK (MAPK), increased expression of LDLR, and fluorescent LDL cholesterol uptake. However, stimulation of MDA-MB-231 (MDA231) cells with EGF did not lead to increased expression of LDLR despite inducing phosphorylation of EGFR. Inhibition of MAPK using UO126 in MDA231 cells reduced LDLR expression, and in MDA468 cells, UO126 impaired the LDLR increase in response to EGF. MDA468 cells exposed to the transcription inhibitor, Actinomycin, prior to treatment with EGF showed reduced degradation of *LDLR* mRNA compared to vehicle-treated cells. Our results suggest that the EGF-associated increase in LDLR protein expression is cell line-specific. The common pathway regulating LDLR expression was MAPK in both TNBC cell lines.

## Introduction

In addition to increased cancer risk and mortality^1,2^, obesity is also linked to dyslipidemia, with increased circulating triglycerides, low-density lipoprotein (LDL) cholesterol, and lower levels of high-density lipoprotein (HDL) cholesterol^3^. There is accumulating evidence that lipid metabolism plays a role in tumor growth^4^; lipids can either be synthesized de novo or taken up from distant sources delivered by circulating lipoproteins. Elevated circulating cholesterol levels have been suggested in preclinical models and clinical studies to be a factor in the promotion of cancer growth^5–8^ and progression^5,9–11^.

Increased lipoprotein uptake is characteristic of some cancers, such as glioblastoma, pancreatic adenocarcinoma and prostate cancer^9–11^. Reduction of LDL receptor (LDLR) expression and LDL uptake, by either enhancing receptor degradation^11^, or genetic manipulation reduced tumor growth in pre-clinical pancreatic and breast cancer models^5,10^, providing evidence for the association between lipoprotein uptake and the promotion of tumor progression in some cancers. Furthermore, in human breast cancer studies, the increased expression of LDLR is associated with shorter recurrence-free survival^5^.

The aim of this study was to understand how LDLR expression is regulated in TNBC. In non-malignant cells, LDLR expression is regulated by multiple factors, including intracellular cholesterol levels, transcription factors such as sterol regulatory element binding proteins (SREBPs), post-transcriptional regulators of mRNA stability, as well as protein degradation^12–15^. Enhanced signaling through the ErbB family of receptors such as epidermal growth factor receptor (EGFR) in glioblastoma and ERBB4 in mammary epithelial and estrogen receptor positive breast cancer cells, has been found to increase LDLR expression through SREBP activation^11,16^ In glioblastoma, hyper-activation of the EGFR signaling pathway led to greater LDLR expression and susceptibility to targeting of cholesterol metabolism^11^. As EGFR is frequently expressed in triple negative breast cancer (TNBC)^17^, we tested whether activation of the EGFR signaling pathway increased LDLR expression in TNBC.

## Results

### EGF stimulates LDL-cholesterol uptake and induces LDLR expression in triple negative breast cancer cells

To determine if the stimulation of the EGFR signaling pathway influences LDLR expression, human breast cancer cell lines were examined for *EGFR* mRNA expression using the Cancer Cell Line Encyclopedia and cBioPortal for Cancer Genomics^18^. The MDA-MB-468 (MDA468) cell line was identified as having the highest *EGFR* mRNA expression compared with other breast cancer cell lines.

**Figure 1:**
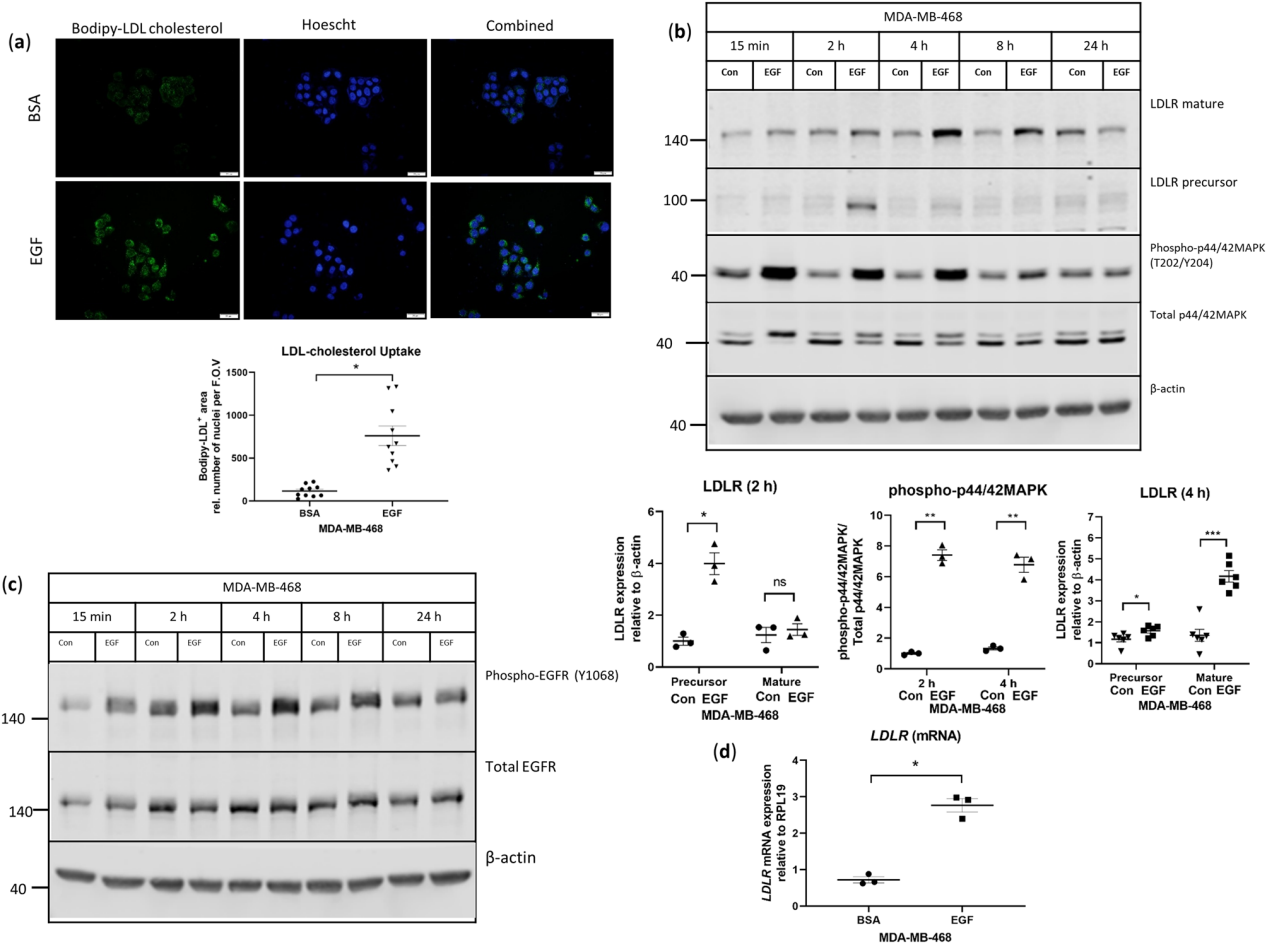
EGF stimulates LDL-cholesterol uptake and induces the expression of LDLR in MDA-MB-468 cells. (**a**) Representative images and quantification of fluorescent LDL-cholesterol uptake by MDA468 cells after stimulation with 10 ng/mL EGF for 5 h, *indicates p = 0.0003 between groups, n = 10 fields of view per group. (**b**,**c**) Representative western blots of LDLR, EGFR and MAPK phosphorylation in MDA468 cells stimulated with 10 ng/mL EGF at the indicated time points. Blots have been cropped for conciseness of presentation and uncropped blots are shown in Supplementary Fig. S7. Quantification of LDLR and phospho-p44/42MAPK are from blots shown in Supplementary Fig. S1 for 2 h and 4 h of EGF stimulation. *Indicates p < 0.05, **indicates p < 0.01, ***indicates p < 0.0001, n = 3 per condition for 2 h and n = 6 per condition for 4 h. (**d**) Gene expression of LDLR in MDA468 cells stimulated with 10 ng/mL EGF for 2 h, *indicates p = 0.003 between groups, n = 3 per condition. Horizontal line indicates mean of the groups, error bars are S.E.M.

To determine if EGF induced the uptake of LDL-cholesterol, we stimulated MDA468 cells with 10 ng/mL EGF and observed increased uptake of fluorescent LDL-cholesterol (Fig. 1a). Stimulation with EGF also led to an increased expression of the partially glycosylated LDLR (precursor) and the fully glycosylated LDLR (mature) (Fig. 1b and Supplementary Fig. S1). Compared with baseline, the LDLR precursor was higher 2 h post-stimulation and mature LDLR expression was greater at 4 h post-stimulation (Fig. 1b). The EGF-stimulated increase of LDLR expression was recapitulated in the murine mammary tumor cell line, Mvt1, which also expresses the EGFR (Supplementary Fig. S2). Increased phosphorylation of EGFR as well as p44/42 MAPK (MAPK), one signaling pathway downstream of EGFR, was also observed in both cell lines (Fig. 1b,c and Supplementary Figs. S1, S2). Stimulation with EGF led to higher levels of *LDLR* mRNA in MDA468 cells (Fig. 1d), suggesting that the increased LDLR protein observed was mediated through an accumulation of *LDLR* mRNA.

### EGF stimulation results in increased LDLR expression through increased stability of LDLR mRNA

**Figure 2:**
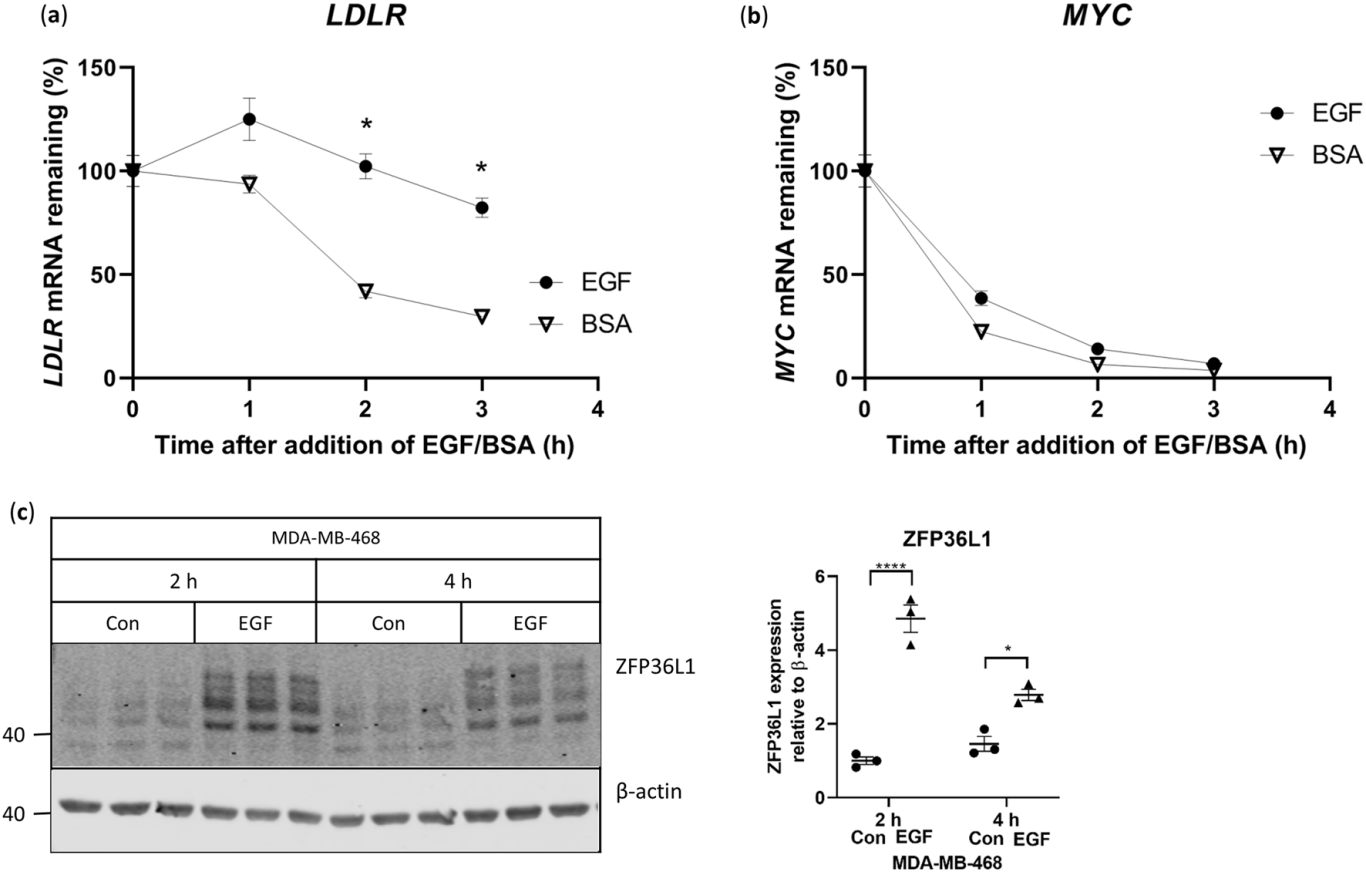
EGF stabilizes *LDLR* mRNA in MDA-MB-468 cells. (**a**,**b**) MDA468 cells were pre-treated with 6.5 μg/mL Actinomycin D for ten minutes prior to stimulation with 10 ng/mL EGF and analyzed by qPCR for gene expression, n = 3 per condition, *indicates p < 0.05 between BSA and EGF at the respective time-point. BSA was added in lieu of EGF as a control. (**c**) Representative western blot of ZFP36L1 in MDA468 cells stimulated with 10 ng/mL EGF for 2 and 4 h. Blots shown are part of the series from Supplementary Fig. S1 and uncropped blots are presented in Supplementary Fig. S4. Note that the same β-actin blot in Supplementary Fig. S1 has been shown here. Brackets indicate groups significantly different. *Indicates p < 0.05 between groups, ****p < 0.0001, as shown.

As *LDLR* mRNA levels were increased with EGF stimulation in the MDA468 cells, we then determined if the EGF-associated effect was mediated by a reduction in mRNA degradation. Accordingly, we examined the rate of *LDLR* mRNA degradation in the presence of the transcription inhibitor, Actinomycin D. Cells were pre-treated with Actinomycin D and then stimulated with EGF, which prevented *LDLR* mRNA degradation (Fig. 2a). To confirm that transcription was fully inhibited, we measured the expression of *MYC* mRNA, which has a short half-life and is inducible with EGF, but is rapidly degraded when transcription is inhibited in the presence of EGF^19^. As shown in Fig. 2b, *MYC* expression decreased with transcription inhibition in both the absence and presence of EGF. These results showed that the EGF-induced increase in *LDLR* mRNA was at least in part due to *LDLR* mRNA protection.

MAPK signaling has previously been linked to the enhancement of *LDLR* mRNA stability through phosphorylation of zinc-finger protein 36 ring finger protein like 1 (ZFP36L1) in HEK293T and HeLa cells, which prevents ZFP36L1-mediated mRNA degradation^20^. As the phosphorylation of MAPK was increased with EGF stimulation, we examined ZFP36L1 phosphorylation in cells treated with EGF. Phosphorylated ZFP36L1 has previously been characterized as a series of bands ranging from 40 to 47 kDa when separated on SDS-PAGE^21^. Exposure of the MDA468 cells to EGF led to higher expression and modification of ZFP36L1 (Fig. 2c) suggesting that the EGF-induced expression of LDLR is mediated through increased MAPK-associated phosphorylation of ZFP36L1, and greater *LDLR* mRNA stability.

### The influence of EGF on LDLR expression is cell line-specific

**Figure 3:**
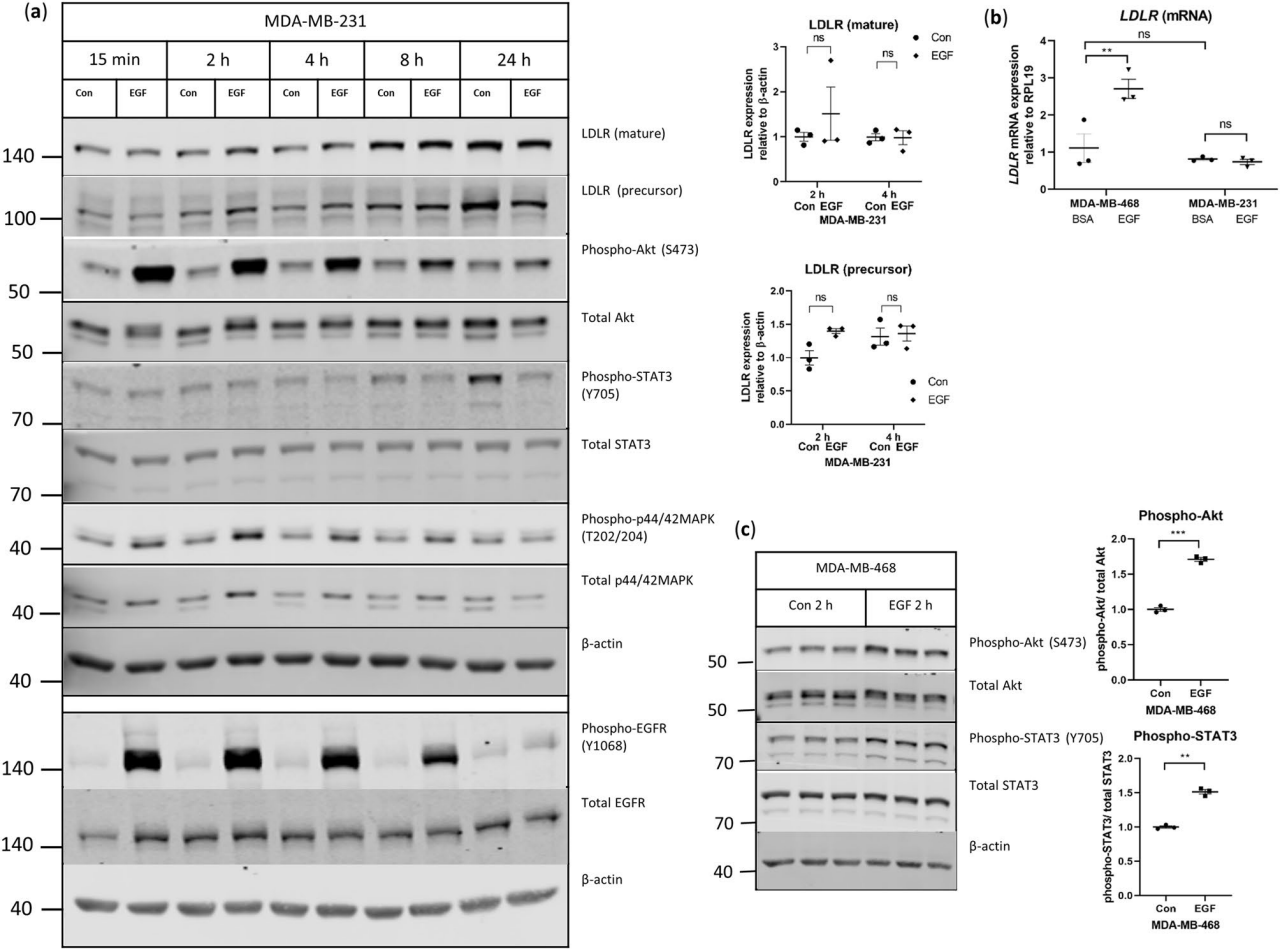
EGF-induction of LDLR is cell line-dependent. (**a**,**b**) Representative western blots of LDLR in MDA231 cells stimulated with 10 ng/mL EGF over 24 h. Two separate gels using the same samples are shown. Uncropped blots are shown in Supplementary Fig. S8. Quantification of LDLR are from blots shown in Supplementary Fig. S3 for 2 h and 4 h of EGF stimulation, n = 3 per condition, “ns” indicates no statistically significant difference between groups. (**b**) Gene expression of *LDLR* in MDA468 and MDA231 cells stimulated with 10 ng/mL EGF for 2 h, n = 3 per condition. **indicates p = 0.006 between groups, “ns” indicates no statistically significant difference between groups, as indicated. (**c**) MDA468 cells were stimulated with 10 ng/mL EGF for 2 h and analyzed by western blotting for protein expression, ***indicates p < 0.0001 between groups for phospho-Akt/total Akt, and **indicates p = 0.0007 between groups for phospho-STAT3/total STAT3, n = 3 per condition. Uncropped blots are shown in Supplementary Fig. S9. Horizontal line indicates mean of the groups, error bars are S.E.M.

In contrast to MDA468 cells, EGF stimulation of MDA231 cells did not result in higher *LDLR* mRNA or protein despite increased phosphorylation of EGFR (Fig. 3a,b, Supplementary Fig. 3), suggesting that the EGF-induction of LDLR is cell line-specific.

We therefore determined if signaling pathways downstream of EGFR activation that have also been previously implicated in LDLR expression, such as Akt^11^ in glioblastoma and STAT3^22^ in prostate cancer, differed in MDA231 (Fig. 3a and Supplementary Fig. 3) and MDA468 cells (Fig. 3c). While stimulation with EGF led to increased phosphorylation of Akt in both cell lines, greater phosphorylation of STAT3 and MAPK were observed only in MDA468 cells (Fig. 3a,c), suggesting that the activation of these pathways might be the basis for the cell line specific difference in LDLR expression in response to EGF.

### STAT3 signaling does not affect LDLR expression in MDA-MB-231 or MDA-MB-468 cells

**Figure 4:**
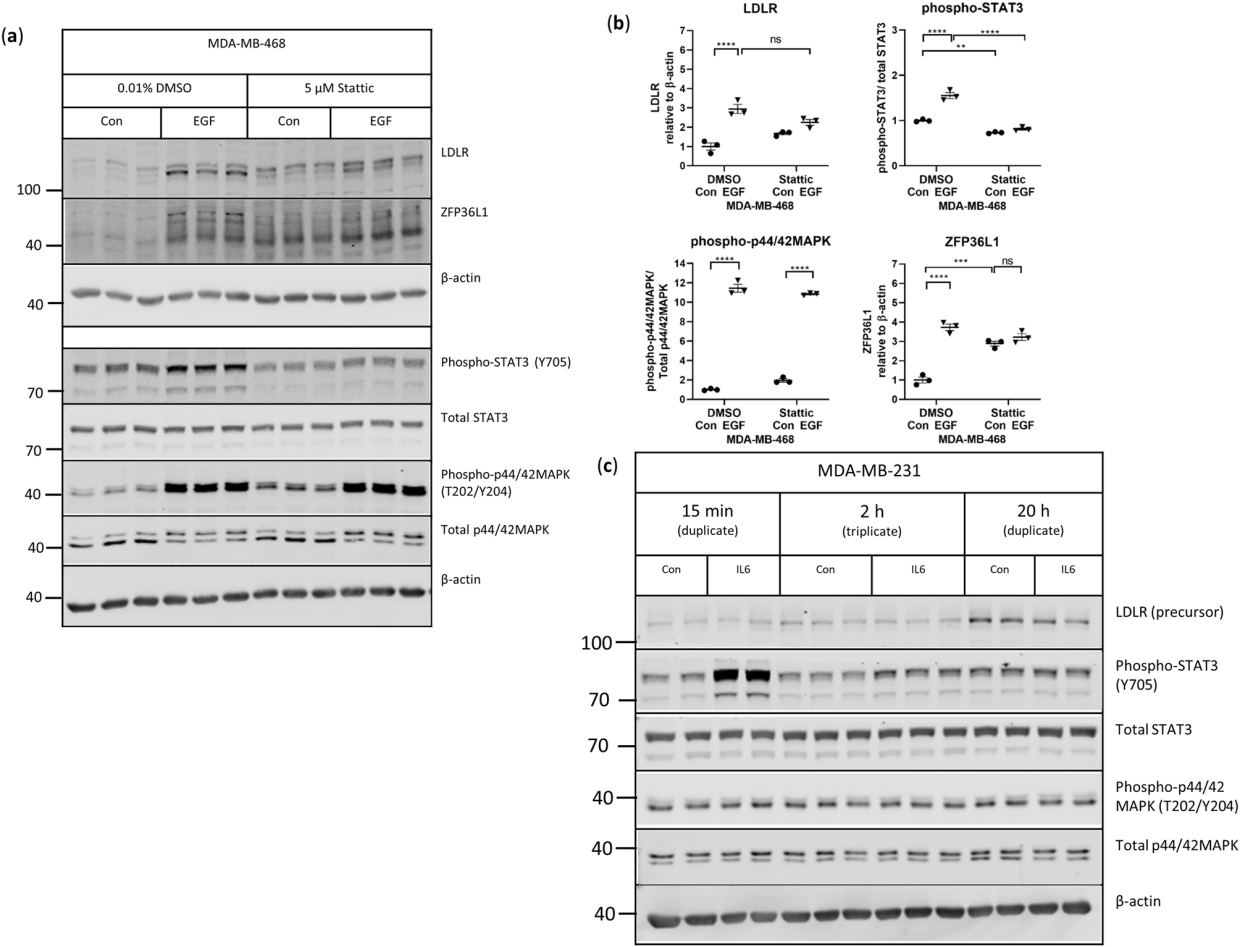
Activation of STAT3 signaling does not affect LDLR expression in MDA-MB-231 cells or in response to EGF in MDA-MB-468 cells. (**a**,**b**) MDA468 cells were pre-treated with 5 µM Stattic for 1 h prior to the addition of BSA (Con) or 10 ng/mL EGF for two hours and analyzed by western blotting for protein expression. Horizontal line indicates mean of the groups, error bars are S.E.M, n = 3 per condition. Brackets indicate groups that are statistically different, *indicates p < 0.05, ***p < 0.001, ****p < 0.0001 between groups, “ns” indicates no statistically significant difference detected between groups, as shown. Uncropped blots are shown in Supplementary Fig. S10. (**c**) MDA231 cells were treated with 20 ng/mL IL-6 for up to 20 h and analyzed by western blotting for protein expression, n = 2–3 per condition. Uncropped blots are shown in Supplementary Fig. S11.

To determine the importance of STAT3 signaling in EGF-induced LDLR expression, we inhibited STAT3 phosphorylation by pre-treating MDA468 cells with a small-molecule inhibitor of STAT3, Stattic. Pre-treatment with Stattic (5 μM) reduced the phosphorylation of STAT3 in response to EGF but did not reduce the EGF-stimulated increase in LDLR expression in MDA468 cells (Fig. 4a,b). However, it was also observed that in the absence of EGF, LDLR expression trended to increase with exposure to Stattic. ZFP36L1 modification was also noted to be greater with exposure to Stattic (Fig. 4a), suggesting that exposure to Stattic at these concentrations induces LDLR expression through the modification of ZFP36L1. Inhibition of STAT3 activation with a different chemical inhibitor, BP-1102, led to a reduction in EGF-induced LDLR expression but demonstrated significant off-target effects, with decreased phosphorylation of Akt and MAPK and modification of ZFP36L1 (data not shown). To determine if STAT3 activation is associated with increased LDLR expression in MDA231 cells, MDA231 were stimulated with 20 ng/mL IL6. Stimulation of MDA231 cells with IL6 resulted in increased levels of phosphorylated STAT3 but had no effect on the phosphorylation of MAPK or LDLR expression (Fig. 4c). Taken together, these observations suggest that activation of STAT3 signaling does not lead to increased expression of LDLR in TNBC, and does not mediate the effect of EGF on LDLR expression in MDA468 cells.

### MAPK signaling is essential for basal LDLR protein expression in MDA-MB-231 cells

**Figure 5:**
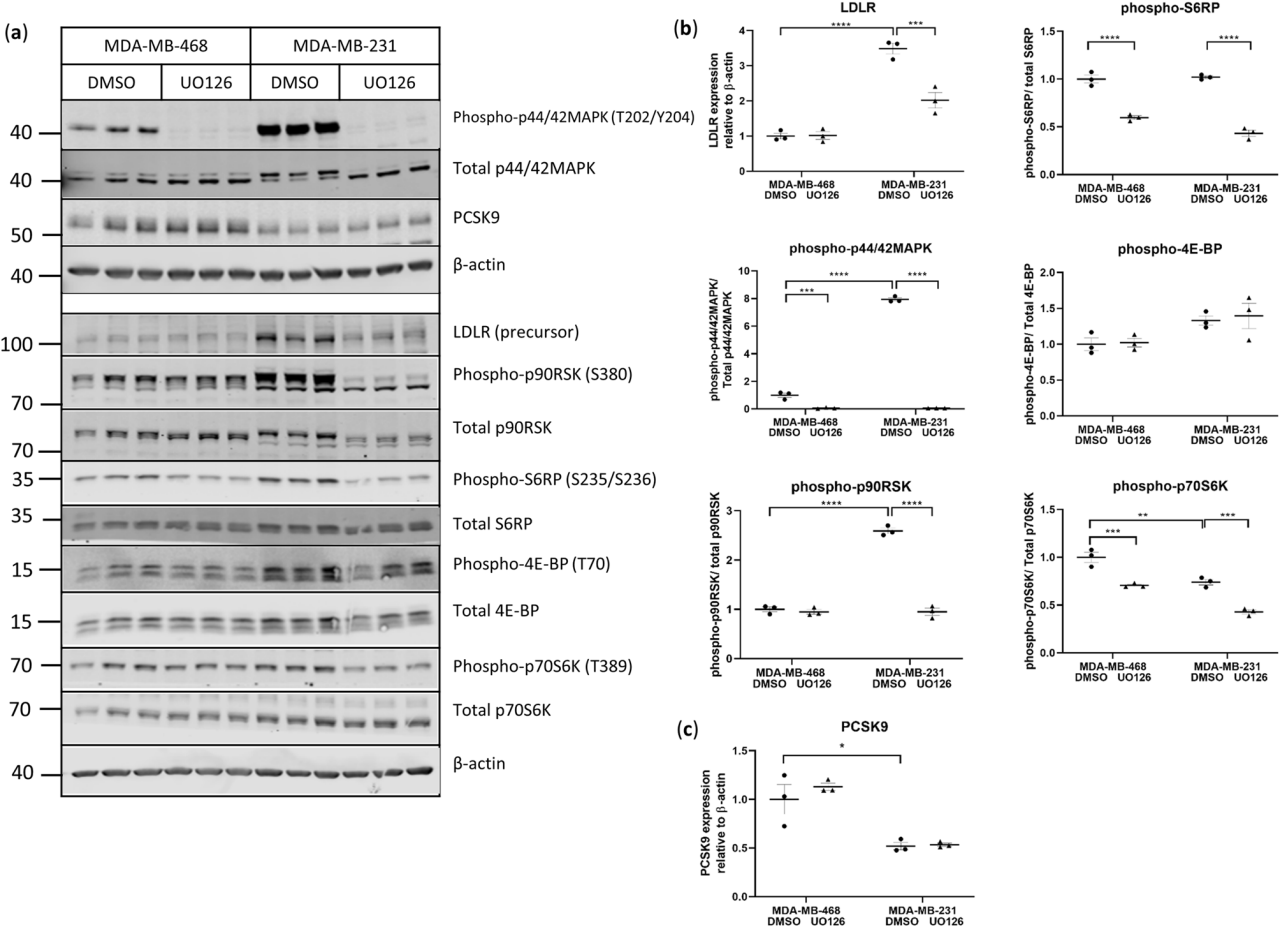
MAPK signaling is important for LDLR expression in MDA-MB-231 cells (**a**,**b**) MDA468 and MDA231 cells were pre-treated with 20 μM UO126 for 1 h prior to the addition of BSA for two hours and analyzed by western blotting for protein expression, n = 3 per condition. Two separate gels using the same samples are shown. Uncropped blots are shown in Supplementary Fig. S12. (**c**) Quantitation of western blot of PCSK9 in MDA468 and MDA231 cells pre-treated with UO126, n = 3 per condition. Horizontal line indicates mean of the groups, error bars are S.E.M. Brackets indicate groups significantly different, *indicates p < 0.05 between groups, **p < 0.01, ***p < 0.001, ****p < 0.0001.

In addition to expressing higher protein levels of LDLR (3.5-fold) in unstimulated conditions, MDA231 cells also showed greater basal phosphorylation of MAPK, and p90 ribosomal S6 kinase (RSK) compared with MDA468 cells (Fig. 5a,b). As the inhibition of MAPK has previously been shown to decrease LDLR mRNA expression in MDA231 cells^23^, we examined pathways downstream of MAPK in unstimulated MDA231 and MDA468 cells treated with the MAPK inhibitor, UO126. In contrast to MDA231 cells in which UO126 reduced LDLR protein expression, LDLR levels remained unchanged in MDA468 cells treated with UO126 in the absence of EGF (Fig. 5a,b). Inhibition of MAPK led to decreased phosphorylation of p70S6K and S6 ribosomal protein (S6RP) in both MDA231 and MDA468 cell lines, but had no effect on phosphorylation of 4E-BP in either (Fig. 5a,b). A reduction in phosphorylated p90RSK was noted in the MDA231, but not MDA468 cells after treatment with UO126 (Fig. 5a,b). These observations confirm that LDLR expression is dependent on MAPK signaling in MDA231 cells and suggest that p90RSK might be a factor downstream of MAPK which mediates this relationship.

Furthermore, MDA468 and MDA231 cells showed similar levels of *LDLR* mRNA at baseline (Fig. 3b) despite differences in protein expression (Fig. 5a,b), suggesting that the differences in LDLR protein expression between the lines are post-transcriptionally mediated. To determine possible underlying mechanisms, the expression of proprotein convertase subtilisin/kexin type 9 (PCSK9), which is associated with LDLR protein degradation, was examined. Protein expression of PCSK9, was lower in MDA231 relative to MDA468 cells and unaffected by MAPK inhibition (Fig. 5a,c), suggesting that LDLR protein levels might be higher in the MDA231 cells in unstimulated conditions because of reduced receptor degradation compared to MDA468 cells.

### MAPK signaling mediates the EGF-stimulated increase in LDLR protein expression in MDA-MB-468 cells

**Figure 6:**
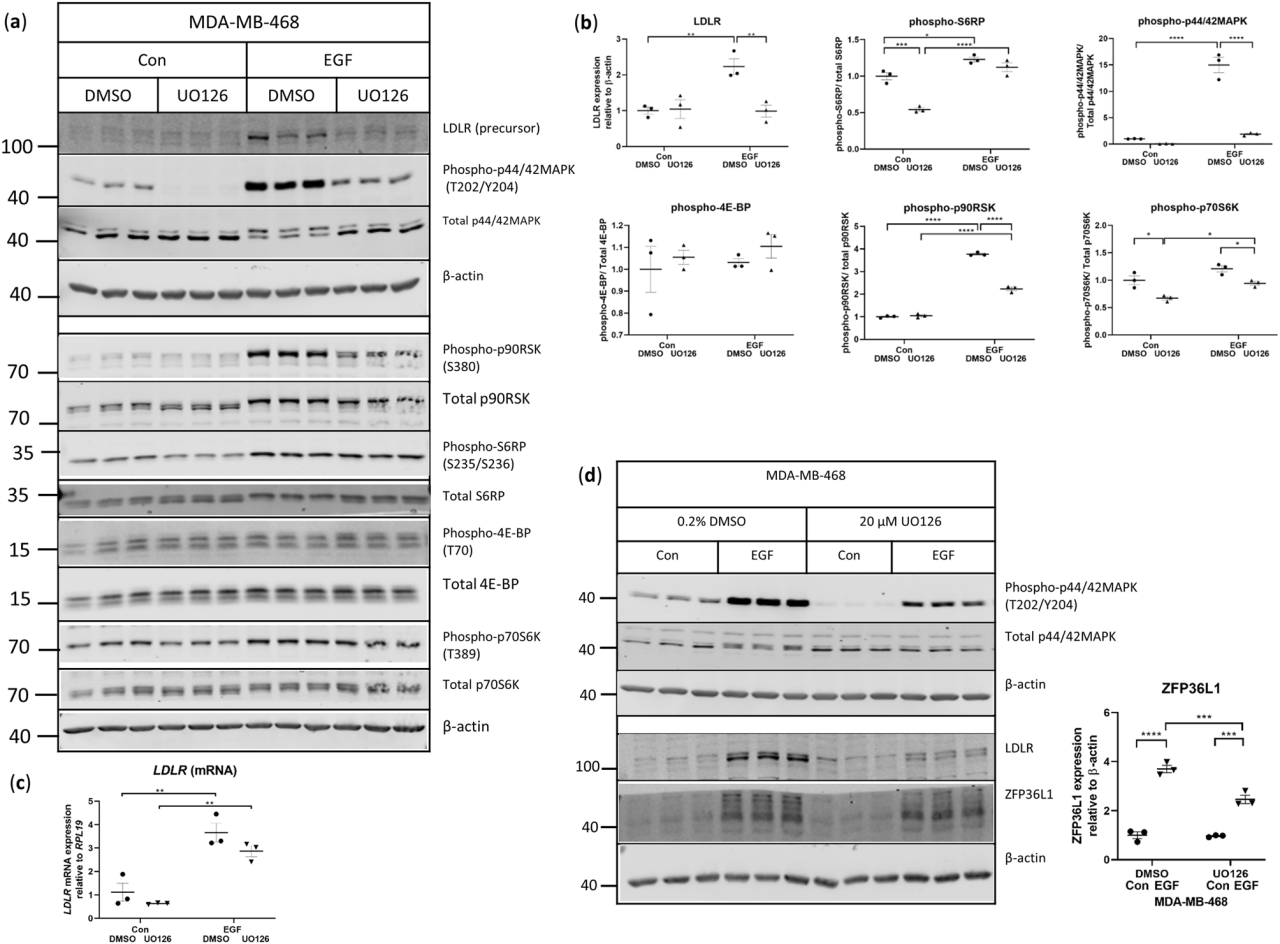
EGF induces the expression of LDLR through the MAPK signaling pathway and stabilizes *LDLR* mRNA in MDA-MB-468 cells. MDA468 cells were pre-treated with 20 μM UO126 for 1 h prior to stimulation with 10 ng/mL EGF for two hours and analyzed by (**a**,**b**) western blotting for protein expression, n = 3 per condition. Two separate gels using the same samples are shown. Uncropped blots are shown in Supplementary Fig. S13. (**c**) mRNA expression of *LDLR* in MDA468 cells treated with UO126 and EGF, n = 3 per condition. (**d**) Representative western blot of ZFP36L1 in MDA468 cells pre-treated with UO126 prior to stimulation with 10 ng/mL EGF. Uncropped blots are shown in Supplementary Fig. S14. Horizontal line indicates mean of the groups, error bars are S.E.M. Brackets indicate groups significantly different, *indicates p < 0.05 between groups, **p < 0.01, ***p < 0.001, ****p < 0.0001.

To determine the importance of MAPK signaling in EGF-induced expression of LDLR, we inhibited EGF-induced MAPK signaling using UO126. Pre-treatment with UO126 reduced EGF-induced phosphorylation of MAPK and LDLR precursor expression in MDA468 cells (Fig. 6a,b). To determine how activation of the MAPK signaling pathway resulted in increased LDLR expression, several proteins downstream of MAPK were assessed. Stimulation with EGF led to higher levels of phosphorylated p90RSK, and S6RP, but no change in phosphorylated levels of p70S6K or 4E-BP were observed (Fig. 6a,b). Only the levels of phosphorylated p90RSK were reduced with MAPK inhibition post-EGF stimulation (Fig. 6a,b).

The EGF-induced increase in *LDLR* mRNA in MDA468 cells trended lower with the inhibition of MAPK signaling (Fig. 6c) and the phosphorylation of ZFP36L1 was also decreased (Fig. 6d), suggesting that the higher expression of *LDLR* mRNA and protein following EGF stimulation is mediated through MAPK signaling. Overall, these results suggest that activation of the MAPK signaling pathway through p90RSK contributes to both the high basal expression of LDLR in MDA231 cells and the EGF-induced increase in LDLR expression in the MDA468 cell line (Fig. 8).

### RSK1/2 signaling is involved in LDLR expression in MDA-MB-468 and MDA-MB-231 cells

**Figure 7:**
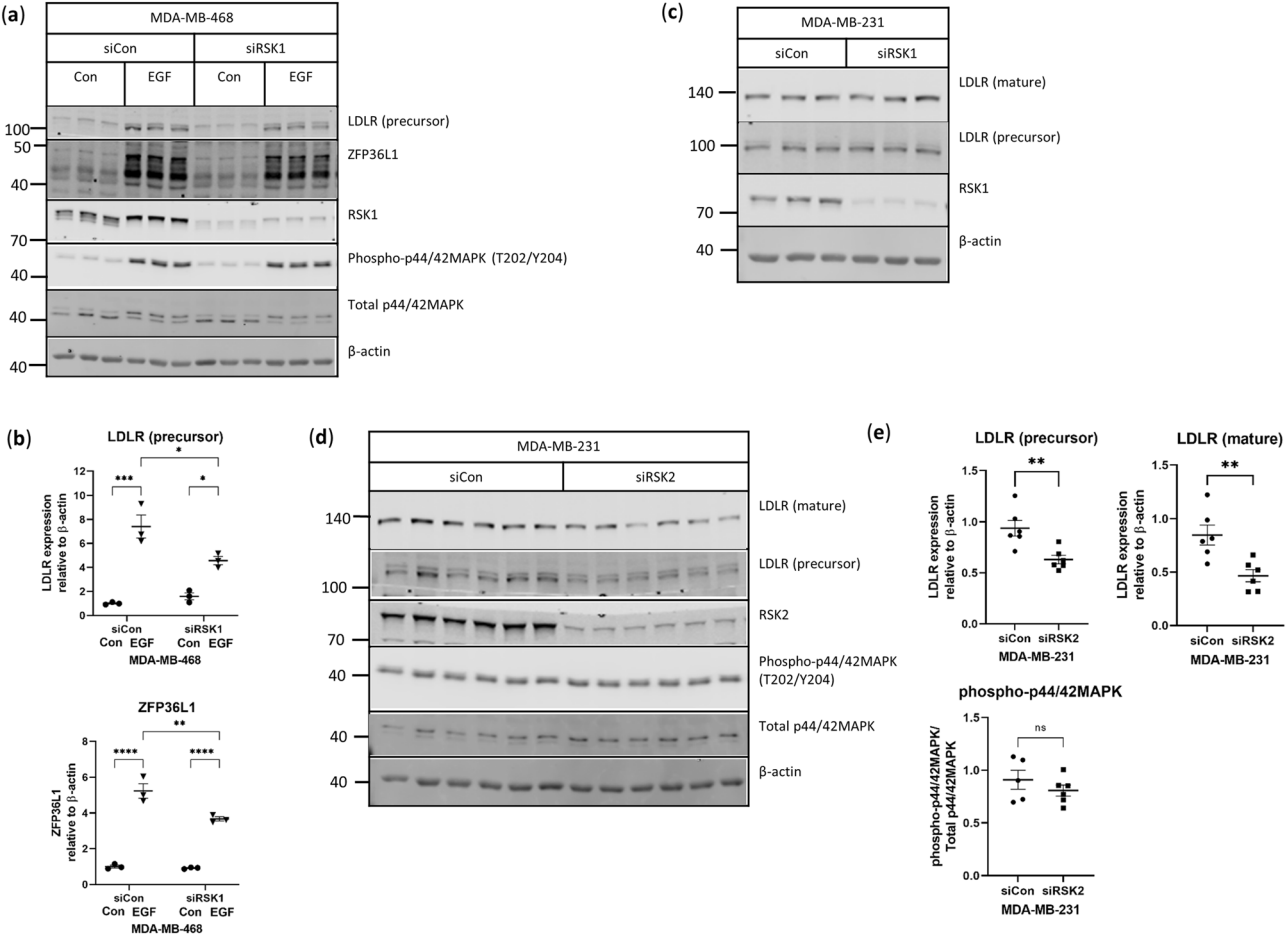
RSK1/2 regulates LDLR expression in MDA-MB-468 and MDA-MB-231 cells. MDA468 cells were transfected with control siRNA or siRNA targeted to RSK1 for 72 h and stimulated with 10 ng/mL EGF for two hours prior to analysis by (**a**,**b**) western blotting for protein expression, n = 3 per condition. Uncropped blots are shown in Supplementary Fig. S15. (**c**) Representative western blot of MDA231 cells transfected with control siRNA or siRNA targeted to RSK1 for 72 h. Uncropped blots are shown in Supplementary Fig. S16. n = 3 per condition. (**d**) Representative western blot of MDA231 cells transfected with control siRNA or siRNA targeted to RSK2 for 72 h. Uncropped blots are shown in Supplementary Fig. S17. n = 6 per condition. Horizontal line indicates mean of the groups, error bars are S.E.M. Brackets indicate groups significantly different, *indicates p < 0.05 between groups, **p < 0.01, ***p < 0.001, ****p < 0.0001.

To confirm that p90RSK mediates the EGF-induced increase in LDLR expression in MDA468 cells downstream of MAPK activation, p90RSK expression was silenced using small interfering RNA (siRNA). In particular, RSK1 was targeted as it has previously been reported to phosphorylate ZFP36L1^20^. The knockdown of RSK1 led to a reduction in the EGF-associated increase in LDLR expression and ZFP36L1 modification in MDA468 cells (Fig. 7a,b), confirming the involvement of RSK1 in EGF-induced LDLR expression. In contrast to the MDA468 cells, knock-down of RSK1 in the MDA231 cells did not lead to a reduction in LDLR expression (Fig. 7c). As MDA231 cells were found to also express RSK2/3 using the Cancer Cell Line Encyclopedia and cBioPortal for Cancer Genomics^18^, RSK2 and RSK3 expression was also silenced via siRNA in MDA231 cells. While the silencing of RSK3 was not observed to have any effect on LDLR expression (data not shown), the silencing of RSK2 led to a reduction in LDLR expression (Fig. 7d,e). Overall our results showed that LDLR expression was regulated by different RSK isoforms in MDA468 and MDA231 cells.

## Discussion

In this study, we observed that activation of the EGFR signaling pathway with EGF led to increased cholesterol uptake and LDLR expression in MDA468 and Mvt1 breast cancer cells. We found that the MAPK signaling pathway downstream of EGFR activation plays a central role in determining LDLR expression through the modulation of *LDLR* mRNA stability in TNBC cells. In previous studies of liver cancer cells, the activation of the EGFR signaling pathway with the EGFR ligands, amphiregulin and epiregulin, led to increased LDLR expression through activation of MAPK signaling and a reduction in *LDLR* mRNA degradation^24^. Similarly, other studies in non-breast cancer cells have linked the activation of the MAPK signaling pathway to increased LDLR expression through reduced mRNA degradation^20,25,26^. Therefore, our results, and previously published studies suggest that the EGFR/MAPK signaling pathway is important for LDLR regulation and a greater susceptibility to the growth-promoting effect of LDL in a number of cancer types.

The timing of activation of p44/42 MAPK and the subsequent increase in *LDLR* mRNA and protein levels in our study are consistent with previous studies, and appear to be cell line and ligand dependent. In HepG2 cells, IL-6 stimulation led to increased *LDLR* mRNA at 2 h, but not 1 h, poststimulation^27^. Similarly the growth factor, neuregulin, increased *LDLR* mRNA at 2 h^16^. The LDLR contains multiple domains and is thought to fold co-translationally with the formation of disulfide bonds, giving rise to a partially glycosylated precursor protein at the endoplasmic reticulum^28,29^. Pulse-chase experiments employing radiolabeled methionine in skin fibroblasts incubated in lipoprotein-deficient FBS, indicated that the LDLR precursor was detectable from 15 min. The mature form was further glycosylated and detected between 30 to 60 min post-pulse^28,30^. The signaling pathway activation, mRNA stabilization by inhibiting the activity of ZFP36L1, requirement for disulfide bond formation and folding during synthesis, followed by extensive glycosylation may explain the timeframe in which we observe the increase in precursor levels 2 h post-EGF stimulation and mature LDLR at a later time-point (4 h post-EGF stimulation).

**Figure 8:**
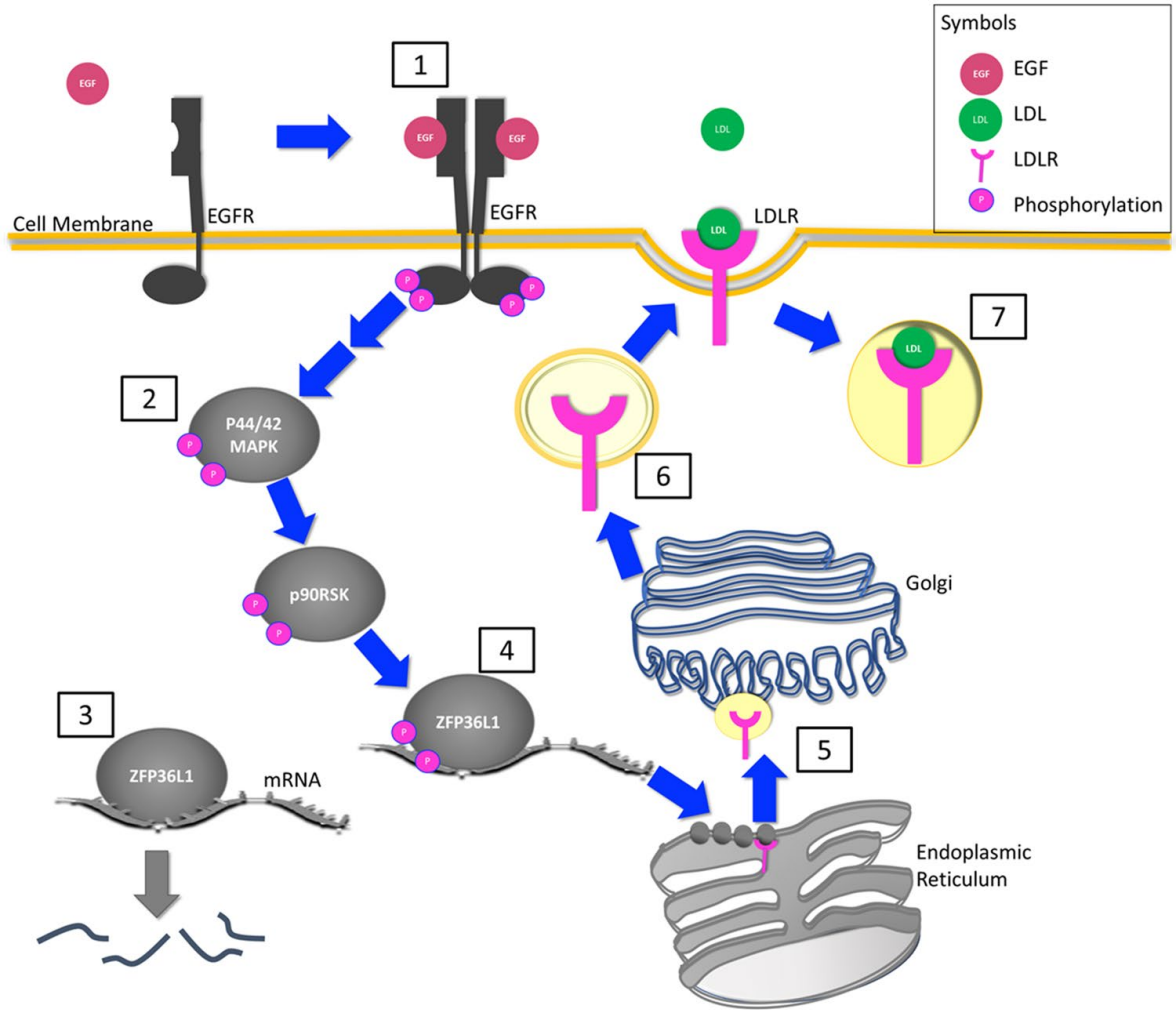
Schematic of proposed mechanism by which EGF stimulation leads to increased LDLR expression. (1) EGF stimulation of the EGF receptor leads to dimerization and phosphorylation of the EGFR. (2) Phosphorylation of the EGFR leads to activation of the MAPK signaling pathway (two arrows indicate intermediaries not shown in the schematic between the EGFR and p44/42 MAPK). Phosphorylation of MAPK leads to activation of p90RSK. (3) ZFP36L1 binds to AU-rich regions within the 3′ untranslated region of *LDLR* mRNA and associates with the deadenylation complex subunit, CNOT7 (not shown in schematic)^20^ leading to RNA degradation. (4) Phosphorylation of ZFP36L1 prevents RNA degradation therefore increasing *LDLR* mRNA levels. (5) LDLR mRNA is translated by the membrane bound ribosomes of the rough endoplasmic reticulum and fully glycosylated in the Golgi. (6) The LDLRs are then transported in vesicles to the cell membrane where they are found in clathrin-coated pits. (7) LDL binds to the LDLR on the cell surface leading to internalization of the LDL/LDLR complex.

The stability of *LDLR* mRNA has been shown to be modulated by multiple RNA-binding proteins, including human antigen R (HuR), KH-type splicing regulatory protein (KSRP), heterogeneous nuclear ribonucleoprotein (hnRNP) I, hnRNPD and ZFP36L1/2. These proteins modulate *LDLR* mRNA expression by binding AU-rich regions within the 3′ untranslated region of *LDLR* mRNA and regulating deadenylation of the poly(A) tail, thereby determining degradation of the mRNA^14,20,31,32^. In particular, the phosphorylation of ZFP36L1/2 by RSK1 downstream of MAPK activation has been shown to result in greater *LDLR* mRNA stability by preventing the association between ZFP36L1 and the deadenylation complex subunit, CNOT7^20^. Our data suggests that activation of the EGFR/MAPK/RSK signaling pathway leads to a modification of ZFP36L1, potentially providing a mechanism by which *LDLR* mRNA stability is increased in MDA468 cells upon EGF stimulation (schematic shown in Fig. 8). ZFP36L1 expression has been reported to play an important role in breast cancer growth and progression by regulating cell cycle progression, hypoxia-associated signaling^33^ and tumor angiogenesis^34^, where intratumoral injection of an active truncated form of ZFP36L1 in a rodent model of breast cancer led to reduced tumor vascular endothelial growth factor expression, vascularization and growth^34^. The role of LDLR/cholesterol homeostasis however, in mediating these effects remains unexplored.

We found that the EGF-induced increase in LDLR is cell line-specific, highlighting the increasingly recognized heterogeneity within TNBCs. In contrast to MDA468 cells, MDA231 cells did not respond with an increase in LDLR mRNA or protein in response to EGF, despite showing increased phosphorylation of EGFR. Other signaling pathways downstream of EGFR activation have previously been reported to be necessary for LDLR expression. The activation of the EGFR/PI3K/Akt signaling pathway has been shown to up-regulate *LDLR* mRNA through SREBP activation in glioblastoma^11^. An increased phosphorylation of Akt was observed in both MDA468 and MDA231 cells with EGF stimulation but did not lead to an increase in LDLR expression in MDA231 cells, suggesting that Akt signaling does not lead to an increase in LDLR expression across cancer cells from different tissues of origin. In contrast, increased phosphorylation of STAT3 was only observed in MDA468 cells with EGF stimulation. By chromatin immunoprecipitation, STAT3 has been shown to play a role in LDLR transcription in prostate cancer, where treatment with 27-hydroxycholesterol resulted in reduced binding of STAT3 to the LDLR promoter, concurrent with a reduction in LDLR expression^22^. Our results based on the use of the chemical inhibitor, Stattic, suggest that STAT3 does not play a role in EGF-induced LDLR expression in TNBC. Interestingly, we observed that treatment with Stattic induced LDLR expression, which is reminiscent of the findings reported with the JAK2 inhibitor, AZD1480. AZD1480 inhibits STAT activity resulting in downregulation of SOCS-3 (a STAT3 target gene), and hyperphosphorylation of Src homology 2 domain-containing protein phosphatase 2 (SHP-2), which resulted in increased p44/42 MAPK phosphorylation in Hodgkin lymphoma cell lines^35^. Enhanced activation of p44/42 MAPK has been described in other cell lines with deletion of SOCS-3^36–38^. We recognize however, that Stattic potentially has non-specific, off-target, effects^39^. Our results suggest that pathways other than Akt and STAT3 account for the cell line-specific response to EGF and highlight the complexity involved in the regulation of *LDLR* mRNA expression.

Contrary to previous findings^18^, we observed that unstimulated MDA468 and MDA231 cells show similar *LDLR* mRNA expression (Fig. 3b). We speculate that the difference in observations may be related to a difference in culture conditions. Nonetheless, we observe greater expression of PCSK9 in the MDA468 cells compared to MDA231 cells, which may indicate increased degradation of LDLR protein, thereby accounting for the reduced LDLR protein observed in MDA468 cells compared to MDA231 cells.

In our studies, inhibition of MAPK signaling in MDA231 cells led to a reduction in LDLR expression, in agreement with previous work by Antalis and colleagues^23^. The phosphorylation of p90RSK, S6RP and p70S6K, which are signaling intermediates downstream of MAPK^40^, were also reduced with MAPK inhibition in our studies. Additionally, we found that silencing RSK2 led to reduced LDLR expression in MDA231 cells. In contrast, unstimulated MDA468 cells, did not decrease LDLR protein expression with MAPK inhibition. It is important however, to note that basal levels of LDLR protein expression are very low in MDA468 cells compared with MDA231 cells. By examining the phosphorylation of p90RSK, S6RP and p70S6K in MDA468 cells stimulated with EGF, we found that only the phosphorylation of p90RSK was increased with EGF stimulation and decreased with MAPK inhibition, suggesting that MAPK signaling increases LDLR expression through p90RSK. Indeed, silencing RSK1 in the MDA468 cells decreased EGF-induced LDLR expression. We also observed that the EGF-induced increase in *LDLR* mRNA trended towards being decreased with MAPK inhibition in MDA468 cells but was not reduced to basal expression levels. This suggests that while MAPK does play a role in increasing *LDLR* mRNA expression post-EGF stimulation, there are other signaling pathways that are likely also contributing to the increase. TNBC has been proposed to be susceptible to the targeting of the MEK/MAPK and PI3K/AKT pathways^41–43^, highlighting the importance of the MAPK signaling pathway in TNBC. To improve response to inhibitors targeting RAS, RAF, MEK, and MAPK, strategies such as the combination of MEK inhibitors with PI3K/Akt^42,43^ and EGFR^44^ inhibitors have been explored in the context of breast cancer. Targeting RSK1 and RSK2 in TNBC has been shown to result in a reduction in tumor growth both in vitro and in murine models^45,46^ and RSK inhibitors such as PMD-026 are currently in clinical trials for metastatic TNBC (NCT04115306).

As the LDLR has previously been demonstrated to be important for hypercholesterolemia-associated breast cancer growth in preclinical models, future studies should explore if targeting MAPK, RSK signaling or ZFP36L1 activity in TNBC provides an additive benefit in the setting of hypercholesterolemia. Human studies are also needed to determine if TNBC’s with activation of the MAPK/RSK pathway have high LDLR expression and are particularly susceptible to the tumor promoting effects of hypercholesterolemia.

The uptake of LDL cholesterol has been shown to enhance cancer progression by supporting tumor cell proliferation and migration^5,9–11,23,47^. In particular, LDLR expression has been associated with poorer outcome in breast cancer^5^. By focusing on the EGFR signaling pathway, where EGFR is more commonly expressed in TNBC^17^, we found that activation of the EGFR signaling pathway led to increased LDLR expression through MAPK/RSK. Overall, our findings identify a key signaling pathway involved in the regulation of LDLR expression, which may be a potential target for the treatment of TNBC in women with obesity.

## Methods

### Cell culture

MDA-MB-468 and MDA-MB-231 cells were obtained from ATCC. Mvt1 cells were previously derived by explant culture of tumors from MMTV c-Myc/Vegf transgenic mice^48^. Human cell line authentication and IMPACT testing of all lines were performed in March 2018 by IDEXX laboratories (Westbrook, MA, USA). For maintenance, cell lines were cultured in DMEM, 10% FBS, penicillin/streptomycin in a humidified incubator with 5% CO_2_ at 37 °C.

Cells were plated and media was switched to DMEM, 1.25% FBS, penicillin/streptomycin, 18 h prior to the start of the experiment. For experiments involving signaling pathway inhibition, cells were pre-treated for 1 h with UO126, Stattic or DMSO, prior to treatment with 10 ng/mL EGF (MilliporeSigma, Burlington, MA, USA) or 0.05% BSA (control). Unless otherwise stated, the concentration of DMSO used for treatment was 0.2% (for UO126 experiments) and 0.01% for Stattic experiments. For investigating RNA degradation, cells were pre-treated with 6.5 μg/mL Actinomycin D (MilliporeSigma), prior to treatment with EGF.

### Western blotting

Protein samples were prepared with lysis buffer (50 mM Tris, 150 mM NaCl, 1 mM EDTA, 1.25% CHAPS, 20 mM NaF, 10 mM Na_2_P_2_O_7_, 8 mM C_3_H_7_Na_2_O_6_P, 1 mM Na_3_VO_4_, supplemented with a protease inhibitor cocktail (Roche, Branchburg, NJ, USA). Proteins were boiled, separated by SDS-PAGE and blotted as previously described^5^. Membranes were imaged using the Li-Cor infrared imaging system and band intensities were quantified using ImageStudio (Li-Cor, Lincoln, NE, USA). The levels of phospho-proteins were quantified and expressed relative to total protein levels. For Akt, p44/42MAPK, STAT3 and S6RP, phospho- and total proteins were probed simultaneously using goat anti-rabbit IRDye 800 W and donkey anti-mouse IRDye 680RD secondary antibodies (Li-Cor). For p90RSK, p70S6K and 4E-BP, blots were probed for phospho-proteins, stripped and re-probed for the respective total protein.

Antibodies and dilutions used were as follows: LDLR (52818, 1:500, Abcam, Cambridge, UK), β-actin (A228, 1:10,000, Sigma-Aldrich, St Louis, MO, USA), phospho-EGFR (Y1068) (3777, 1:1000, Cell Signaling Technology (CST), Danvers, MA, USA), Total EGFR (for human cell lines) (CST4267, 1:1000), Total EGFR (Mvt1) (CST2646, 1:1000), phospho-STAT3 (Y705) (CST9145, 1:2000), Total STAT3 (CST9139, 1:1000), phospho-Akt (S473) (CST9271, 1:1000), Total Akt (CST2920, 1:2000), phospho-p44/42MAPK (T202/Y204) (CST9101, 1:1000), Total p44/42MAPK (CST9107, 1:1000), ZFP36L1 (CST2119, 1:1000), phospho-p70S6K (T389) (CST9234, 1:1000), Total p70S6K (CST2708, 1:1000), phospho-S6RP (S235/236) (CST2211, 1:1000), Total S6RP (CST2317, 1:1000), phospho-4EBP (T70) (CST9455, 1:1000), Total 4EBP (CST9644, 1:1000), phospho-p90RSK (Ser380) (CST11989, 1:1000), Total RSK (CST9355, 1:1000), RSK1 (CST8408, 1:1000), RSK2 (CST5528, 1:1000).

### Quantitative PCR

Quantitative PCR was performed using Sybr Green (Qiagen, Germantown, MD, USA) on samples that were isolated with TRIzol (Life Technologies, Carlsbad, CA, USA) and purified using RNeasy Mini Kit spin columns (Qiagen) by following the manufacturer’s instructions. Primers used: LDLR FWD: GGTCTGACCTGTCCCAGAGA, LDLR REV: CCTGGATGTCTCTGCTGATG, PCSK9 FWD: ACCCTCATAGGCCTGGAGTT, PCSK9 REV: GAGTAGAGGCAGGCATCGTC, RPL19 FWD: AGCTCTTTCCTTTCGCTGCT, RPL19 REV: GATCTGCTGACGGGAGTTGG, MYC FWD: CTCCTGGCAAAAGGTCAGAG, MYC REV: TCGGTTGTTGCTGATCTGTC.

### Fluorescent LDL uptake

MDA-MB-468 cells were grown on coverslips and growth media was switched to 1.25% FBS overnight prior to the start of the assay. Cells were treated with 10 ng/mL EGF or 0.05% BSA (control) for 5 h, with 10 μg/mL Bodipy LDL-cholesterol (L3483, Life technologies) added for the final 1 h at 37° in the dark. Nuclei were stained with Hoescht (R37605, Life Technologies) prior to rinsing the cells with ice-cold PBS + 0.5% BSA twice and fixing with 4% paraformaldehyde. Coverslips were mounted with aqueous medium. Images were acquired using an Olympus AX70 microscope with 40× objective. Optimal exposure time was determined using the negative controls and maintained for both EGF- and control-treated cells. 5 fields-of-view were acquired per biological replicate. Images in TIFF format were quantified using CellProfiler^49^.

### siRNA-mediated gene silencing

MDA-MB-468 or MDA-MB-231 cells were transfected using DharmaFECT 1 transfection reagent (Dharmacon, Lafayette, CO) with either non-targeting SMARTpool siRNA or ON-TARGETplus SMARTpool siRNA(s) targeting either RPS6KA1, RPS6KA2 or RPS6KA3 (Dharmacon). Cells were plated in antibiotic-free medium containing 10% FBS and transfected the next day with 25 nM siRNA. The culture medium was switched to 1.25% FBS 18 h prior to harvest. Harvest was conducted 72 h post-transfection. For experiments involving EGF, 10 ng/mL EGF was added 2 h prior to harvest.

### Statistical analysis

All results are reported as the group mean ± S.E.M. Power analyses were performed for experiments to determine sample size. Experiments were performed as two or three independent experiments with biological replicates, as indicated. Data that were normally distributed were analyzed by parametric tests (Student’s 2 tailed t-test for two groups, and ANOVA for more than two groups, with posthoc Tukey’s test), or non-parametric tests (the Mann–Whitney U test or Kruskall Wallis H test, as appropriate) where data were not normally distributed. Data analysis was performed using GraphPad Prism v.8 (GraphPad Software, San Diego, CA). A p-value < 0.05 was considered statistically significant.

## Supplementary Information

Supplementary Figures.

## Data Availability

All data generated or analyzed during for this study are included in the published article and its Supplementary information file.
