# Supplementary material for: Regulation of low-density lipoprotein receptor expression in triple negative breast cancer by EGFR-MAPK signaling
Source: Sci Rep. 2021 Sep 9;11:17927. doi: 10.1038/s41598-021-97327-y (PMC8429745; doi:10.1038/s41598-021-97327-y)
Supplement: Supplementary file 1 — Supplementary Figures. [file 41598_2021_97327_MOESM1_ESM.pdf]

Title: Regulation of low-density lipoprotein receptor expression in triple negative breast cancer by EGFR-MAPK signaling

Authors: Tiffany Scully[1], Nathan Kase[1], Emily Jane Gallagher[1,2,#,*], Derek LeRoith[1,2,#]

Authors' Institutions / Addresses:
1. Division of Endocrinology, Diabetes and Bone Disease, Icahn School of Medicine at Mount Sinai, One Gustave L. Levy Place, New York, NY 10029, USA; **2.** Tisch Cancer Institute at Mount Sinai, Icahn School of Medicine at Mount Sinai, One Gustave L. Levy Place, New York, NY 10029, USA; [#]These individuals are co-senior authors.

Corresponding Author: Emily J. Gallagher, M.D., Ph.D., Division of Endocrinology, Diabetes and Bone Disease, One Gustave L. Levy Place, Box 1055, New York, NY 10029, USA. **Phone:** 212-241-1500; **Email:** Emily.Gallagher@mssm.edu

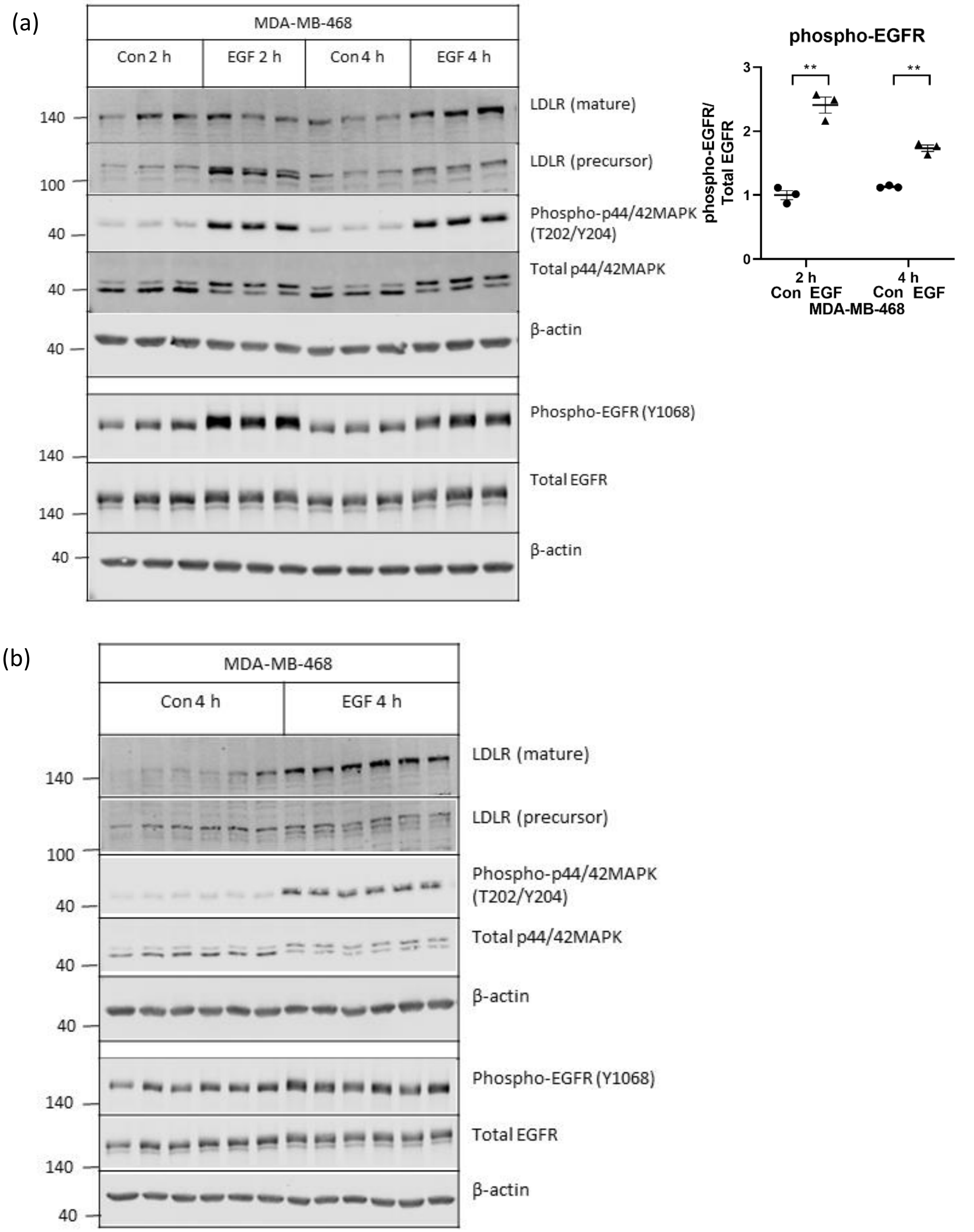

Supplementary Figure 1: EGF stimulates LDLR expression in MDA-MB-468 cells. MDA-MB-468 cells were stimulated with 10 ng/mL EGF for 2 and 4 hours and analysed by western blotting for protein expression. Quantification for these Western blots are also in Figure 1. ** indicates $p < 0.01$. (a) n = 3 per condition, (b) n = 6 per condition. Uncropped blots are presented in Suppl. Fig. S4.

(a

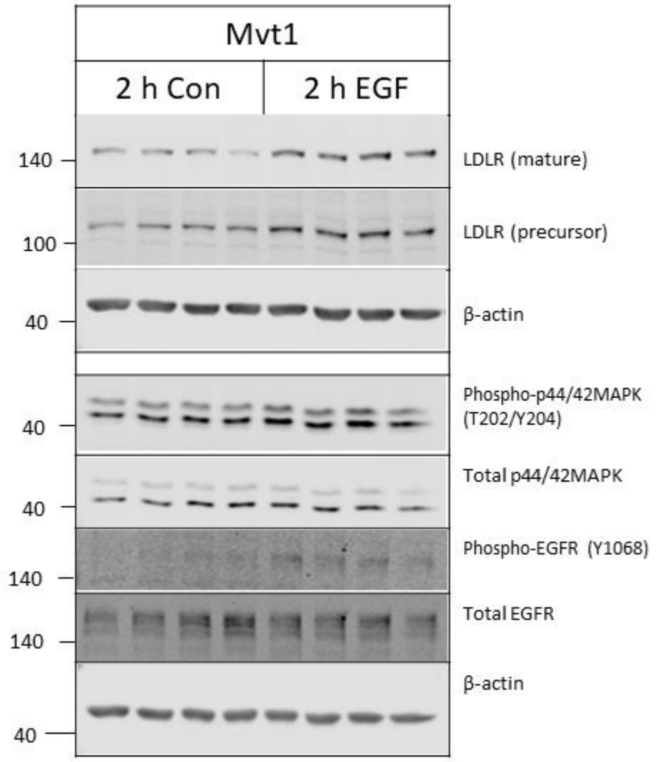

(b)

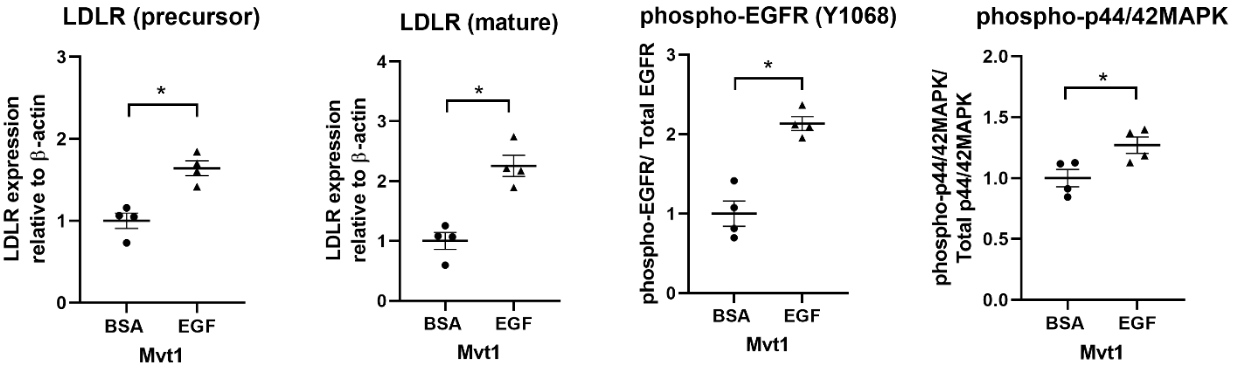

Supplementary Figure 2: EGF stimulates LDLR expression in Mvt1 cells. Mvt1 cells were stimulated with 10 ng/mL EGF for 2 hours and analysed by western blotting for protein expression. *$p < 0.05$, n = 4 per condition. Data are presented as means ± S.E.M. Uncropped blots are presented in Supp. Fig. S5.

Supplementary Figure S3

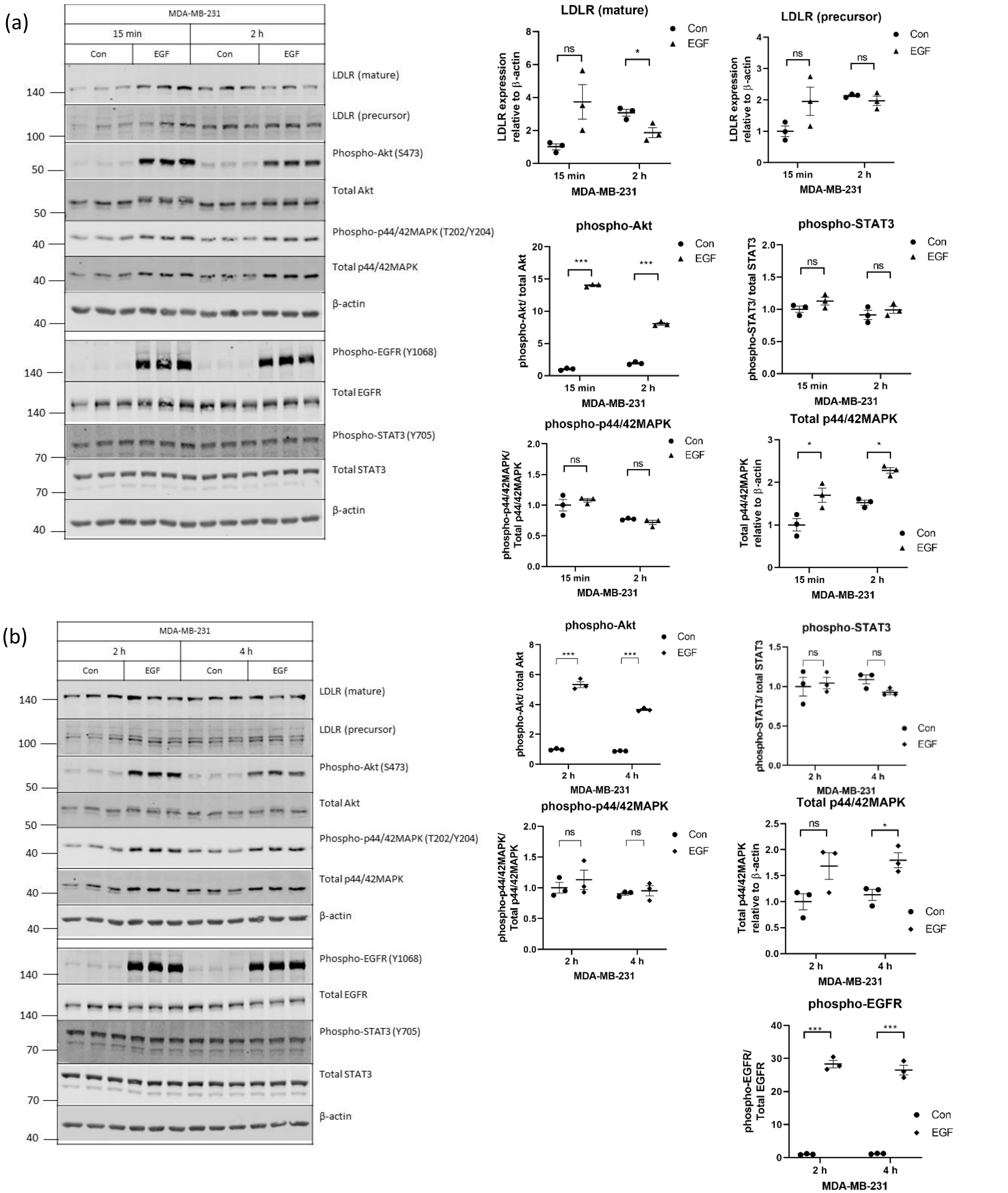

Supplementary Figure 3: EGF does not stimulate LDLR expression in MDA-MB-231 cells. MDA-MB-231 cells were stimulated with 10 ng/mL EGF for (a) 15 minutes and 2 hours or (b) 2 and 4 hours and analysed by western blotting for protein expression. Quantification for LDLR in (b) is shown in Figure 3(a). n = 3 per condition, $*p < 0.05$, $***p < 0.0001$, ns = non-significant. Data is presented as means ± S.E.M. Uncropped blots are presented in Supp. Fig. S6a, S6b.

Supplementary Figure S4a

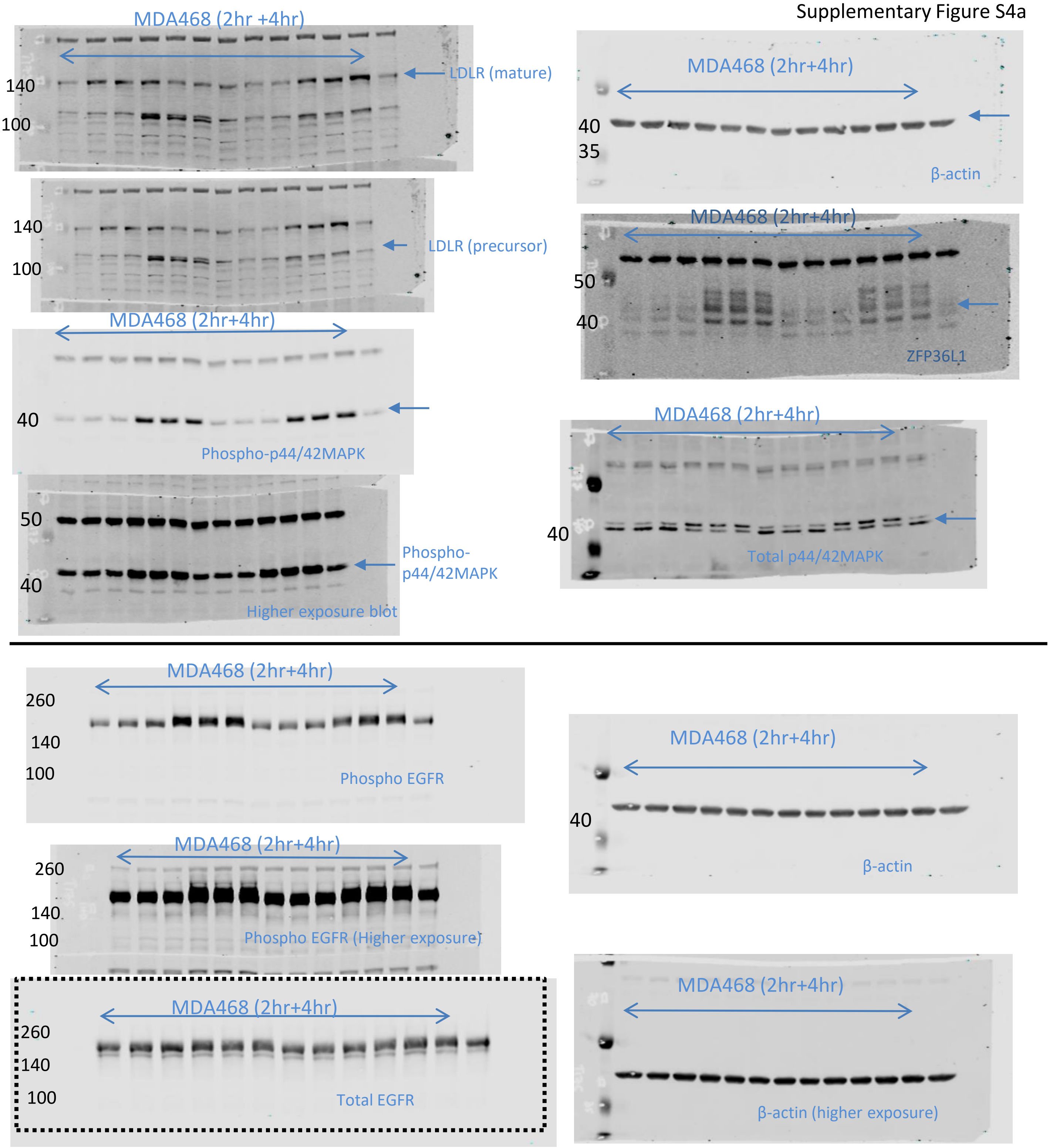

Supplementary Figure 4a: Uncropped version of blots presented in both S1(a) and Fig.2(c). Two separate blots were generated using the same samples. Top blot: The membrane was cut below 100 kDa to blot for LDLR and phospho-p44/42MAPK. Phospho-p44/42MAPK (rabbit source) and total p44/42MAPK (mouse source) probing were done concurrently using secondary antibodies conjugated to different fluorochromes. The membrane was then, stripped and re-probed for beta-actin, followed by stripping and re-probing for ZFP36L1. Bottom blot: The blot was cut below 100 kDa to probe for phospho-EGFR and beta-actin. The membrane was then stripped and re-probed for total EGFR. Blue arrows indicate the location of the bands-of-interest. Multiple exposures are shown for blots that were either taken with a higher contrast or that do not have backgrounds sufficiently dark to show the membrane periphery. Dotted lines indicate membrane periphery.

Supplementary Figure S4b

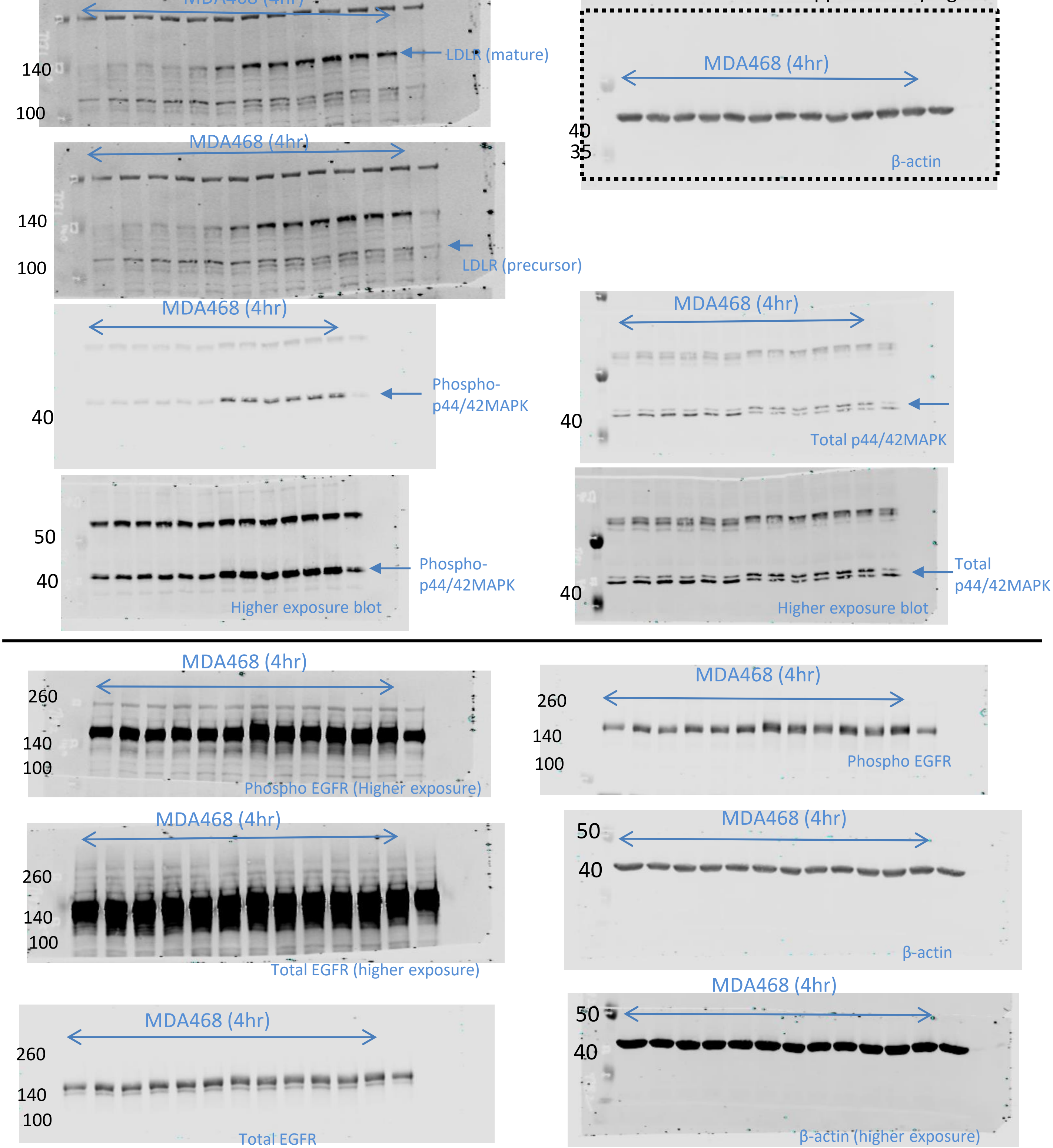

Supplementary Figure 4b: Uncropped version of blots presented in S1(b). Two separate blots were generated using the same samples. Top blot: The membrane was cut below 100 kDa to blot for LDLR and p44/42 MAPK. phospho-p44/42MAPK (rabbit source) and total p44/42MAPK (mouse source) probing were done concurrently using secondary antibodies conjugated to different fluorochromes. The membrane was stripped and re-probed for beta-actin. Bottom blot: The membrane was cut below 100 kDa to probe for phospho-EGFR and beta-actin. The membrane was then stripped and re-probed for total EGFR. Blue arrows indicate the location of the bands-of-interest. Multiple exposures are shown for blots that were either taken with a higher contrast or that do not have backgrounds sufficiently dark to show the membrane periphery. Dotted lines indicate membrane periphery.

Supplementary Figure S5

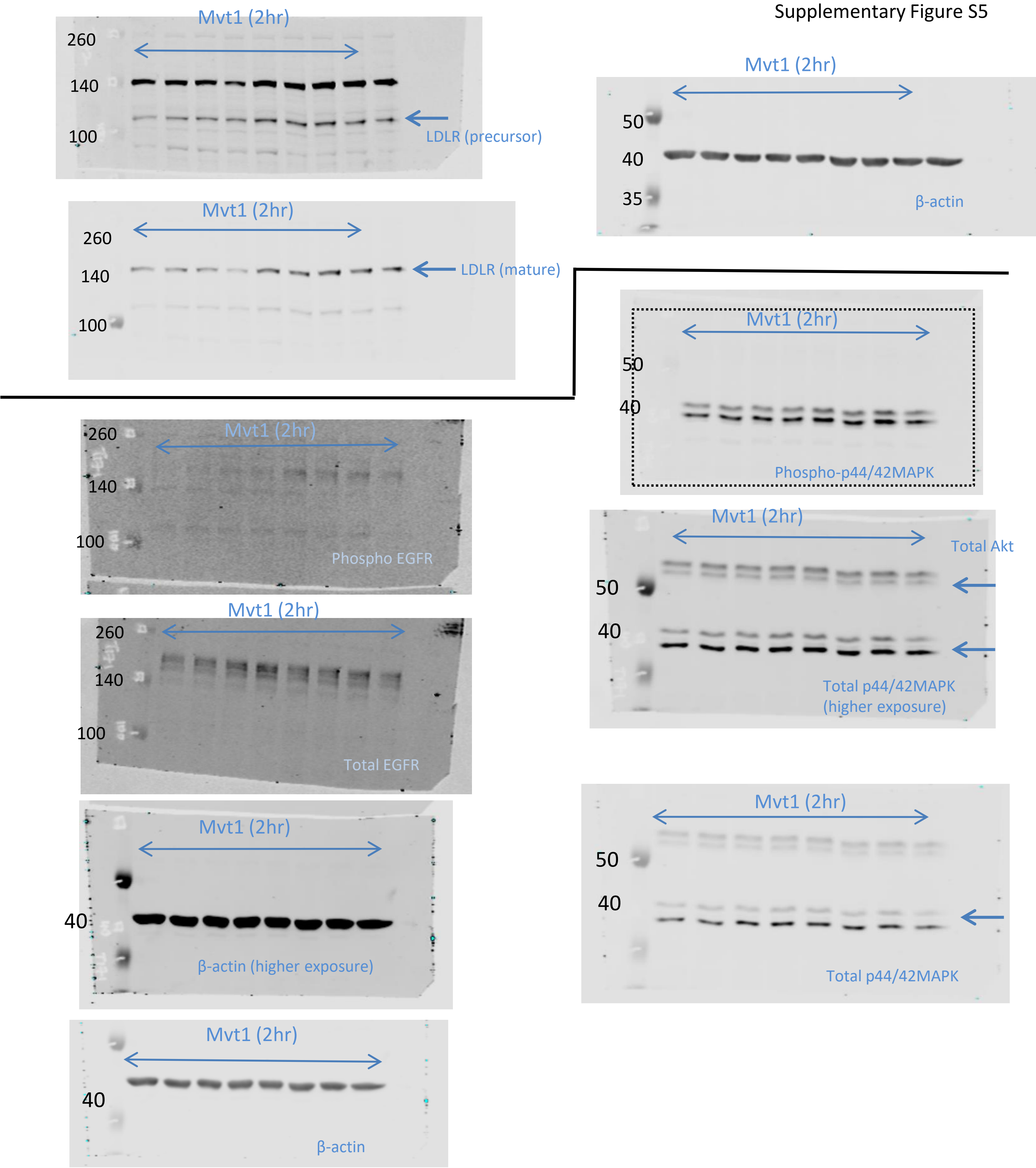

Supplementary Figure 5: Uncropped version of blots presented in Figure S2. Two separate blots were generated using the same samples. Top blot: The membrane was cut below 100 kDa to blot for LDLR and beta-actin. Bottom blot: The membrane was cut below 100 kDa to probe for phospho-EGFR, p44/42MAPK and Akt. The membrane was then stripped and re-probed for total EGFR and beta-actin. phospho-p44/42MAPK (rabbit source) and total p44/42MAPK (mouse source) probing were done concurrently using secondary antibodies conjugated to different fluorochromes. Blue arrows indicate the location of the bands-of-interest. Multiple exposures are shown for blots that were either taken with a higher contrast or that do not have backgrounds sufficiently dark to show the membrane periphery. Dotted lines indicate membrane periphery.

Supplementary Figure S6a

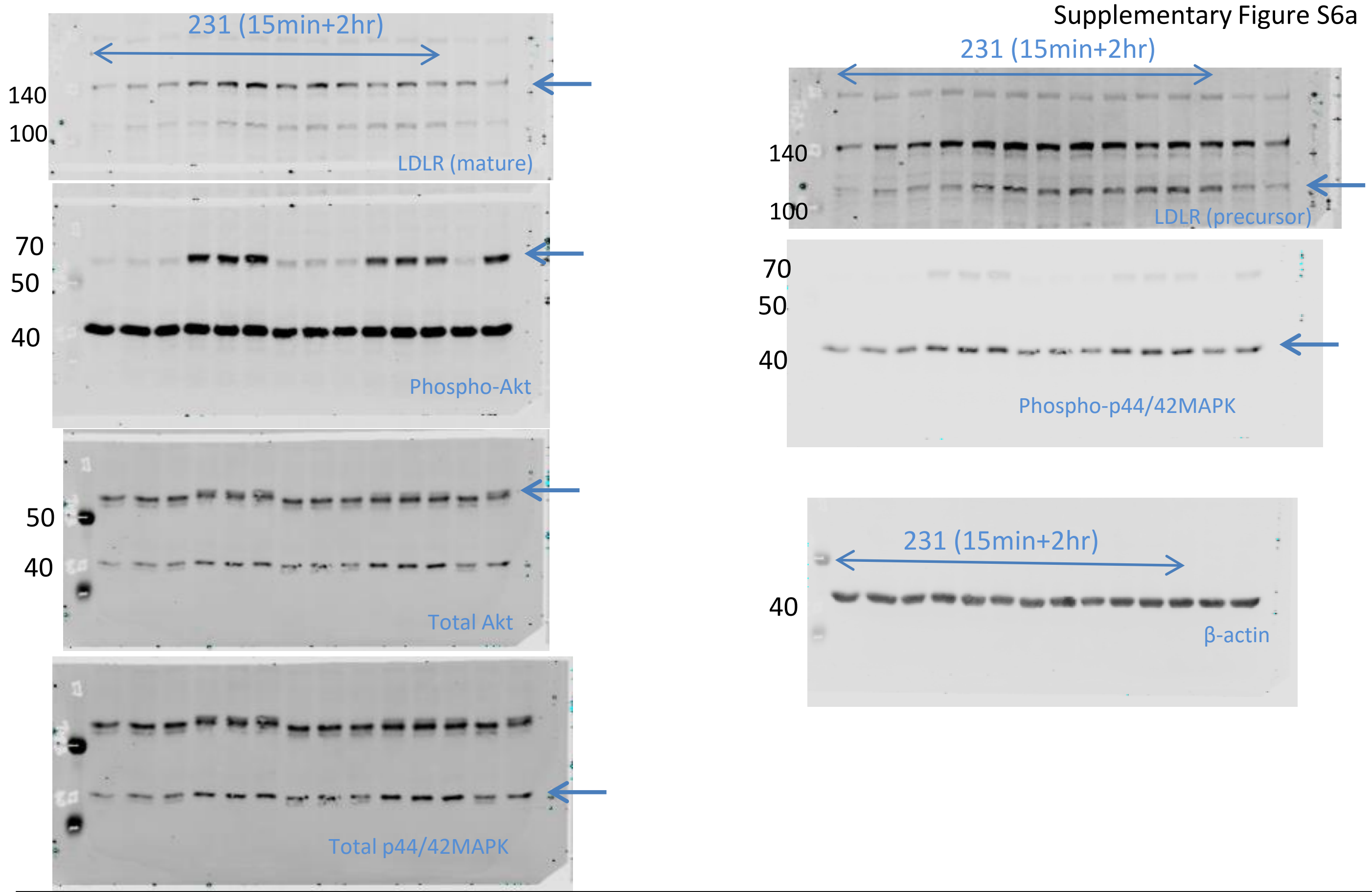

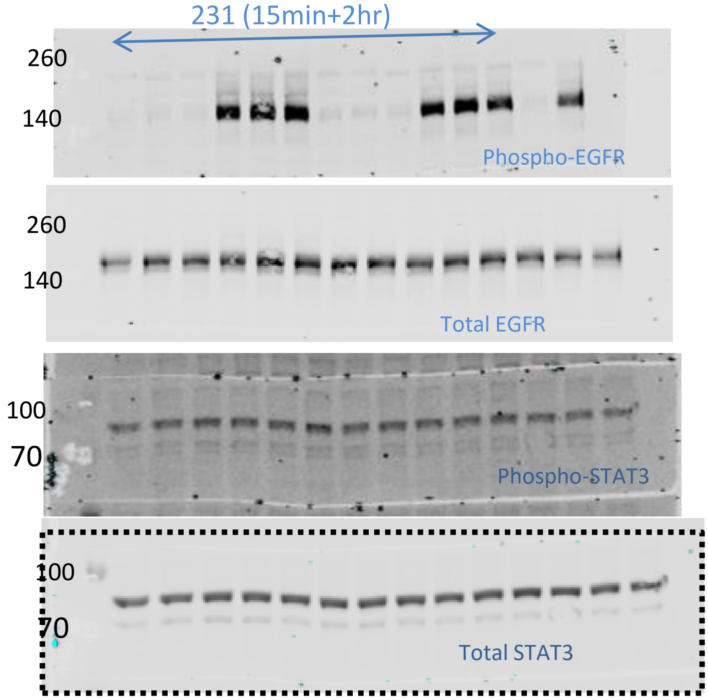

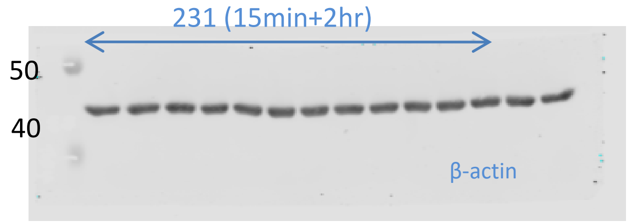

Supplementary Figure 6a: Uncropped version of blots presented in S3(a). Two separate blots were generated using the same samples. Top blot: The membrane was cut above 70 kDa to blot for LDLR, Akt and p44/42MAPK. phospho-p44/42MAPK or phospho-Akt (rabbit source) and total p44/42MAPK or total Akt (mouse source) probing were done concurrently using secondary antibodies conjugated to different fluorochromes. The membrane was then stripped, and re-probed for beta-actin. Bottom blot: The membrane was cut above 100 kDa to probe for phospho-EGFR and STAT3. The membrane was then stripped and re-probed for total EGFR and beta-actin. phospho-STAT3 (rabbit source) and total STAT3 (mouse source) probing were done concurrently using secondary antibodies conjugated to different fluorochromes. Blue arrows indicate the location of the bands-of-interest. Dotted lines indicate membrane periphery.

Supplementary Figure S6b

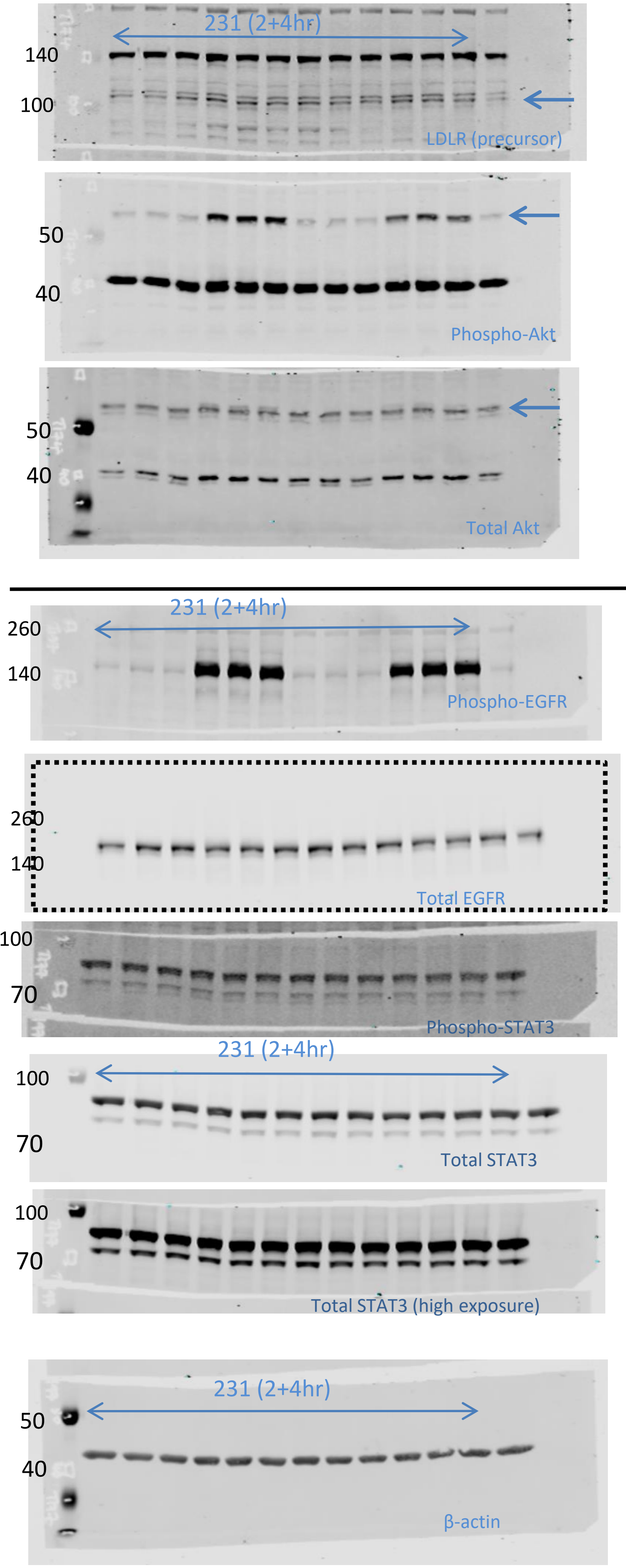

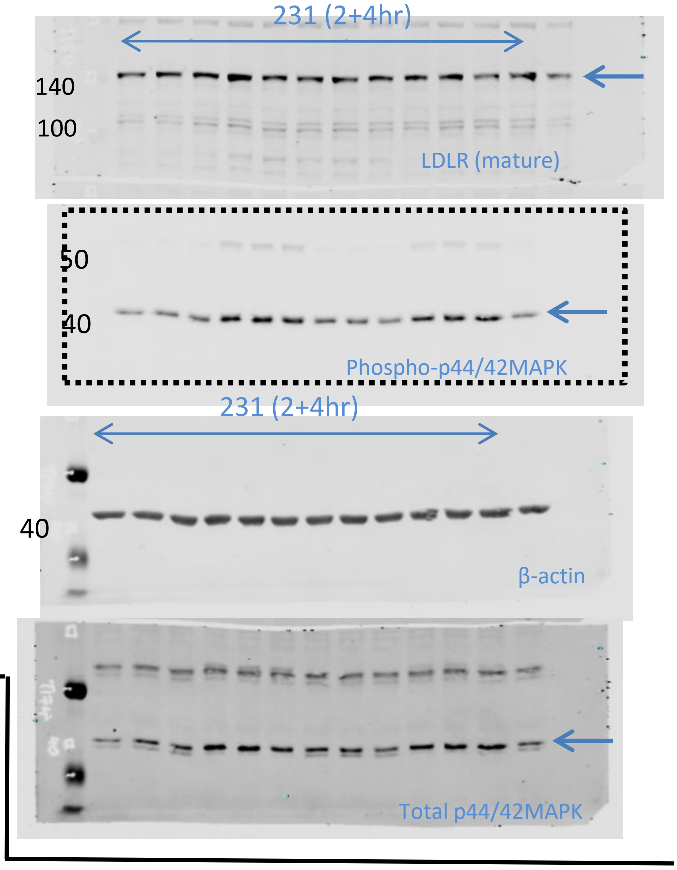

Supplementary Figure 6b: Uncropped version of blots presented in S3(b). Two separate blots were generated using the same samples. Top blot: The membrane was cut above 70 kDa to blot for LDLR, Akt and p44/42MAPK. phospho-p44/42MAPK or phospho-Akt (rabbit source) and total p44/42MAPK or total Akt (mouse source) probing were done concurrently using secondary antibodies conjugated to different fluorochromes. The membrane was then stripped, and re-probed for beta-actin. Bottom blot: The blot was cut above 100 kDa to probe for phospho-EGFR and STAT3. The blot was then stripped and re-probed for total EGFR and beta-actin. phospho-STAT3 (rabbit source) and total STAT3 (mouse source) probing were done concurrently using secondary antibodies conjugated to different fluorochromes. Blue arrows indicate the location of the bands-of-interest. Multiple exposures are shown for blots that were either taken with a higher contrast or that do not have backgrounds sufficiently dark to show the membrane periphery. Dotted lines indicate membrane periphery.

Supplementary Figure S7a: Figure 1b

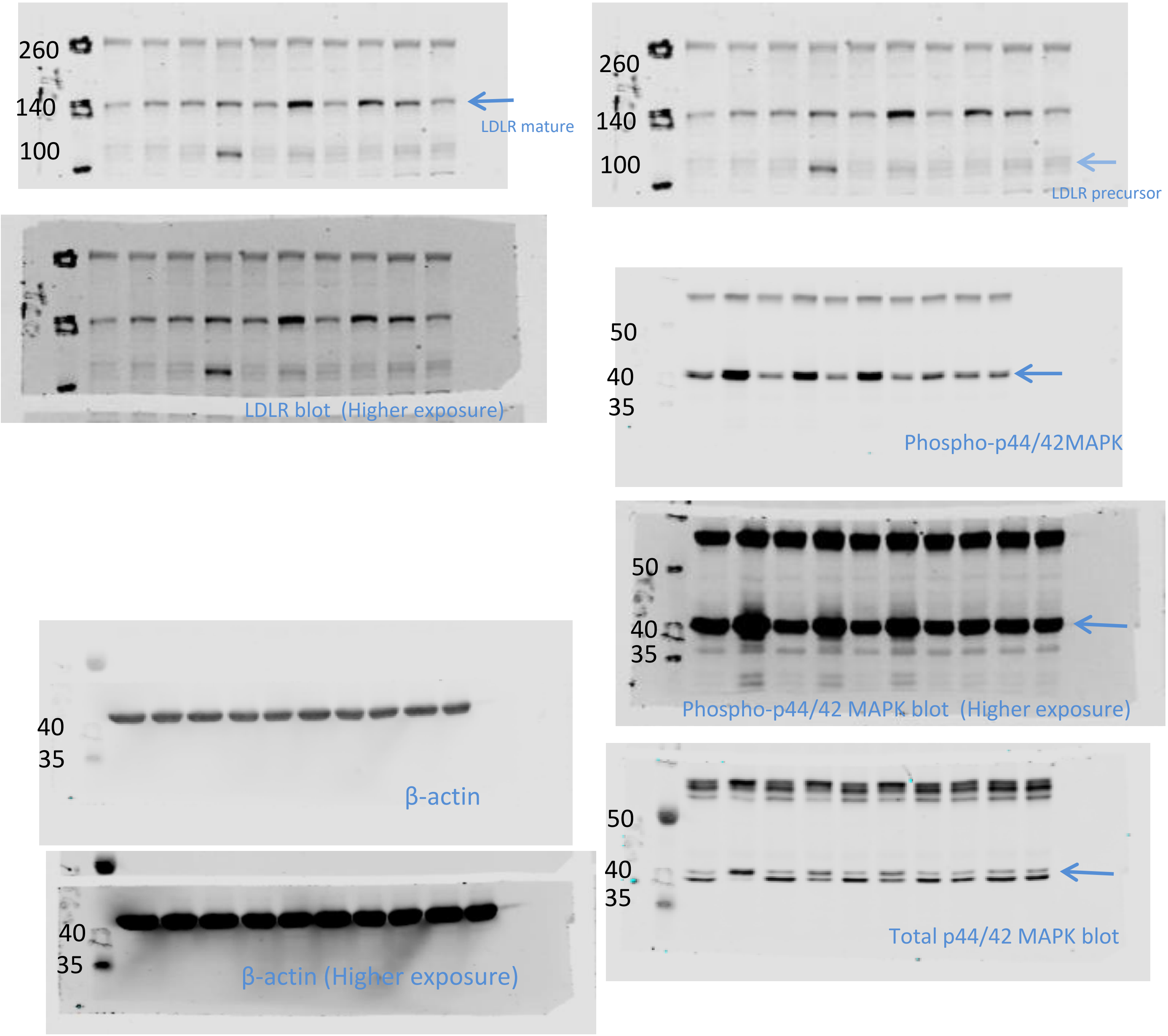

Supplementary Figure 7a: Uncropped version of blots presented in main Figure 1(b). The fresh membrane was cut below 100 kDa to blot for LDLR and MAPK. Phospho-MAPK (rabbit source) and total MAPK (mouse source) probing were done concurrently using secondary antibodies conjugated to different fluorochromes. The membrane was stripped and re-probed for β-actin. Blue arrows indicate the location of the bands-of-interest. Multiple exposures are shown for blots that were either taken with a higher contrast or that do not have backgrounds sufficiently dark to show the membrane periphery.

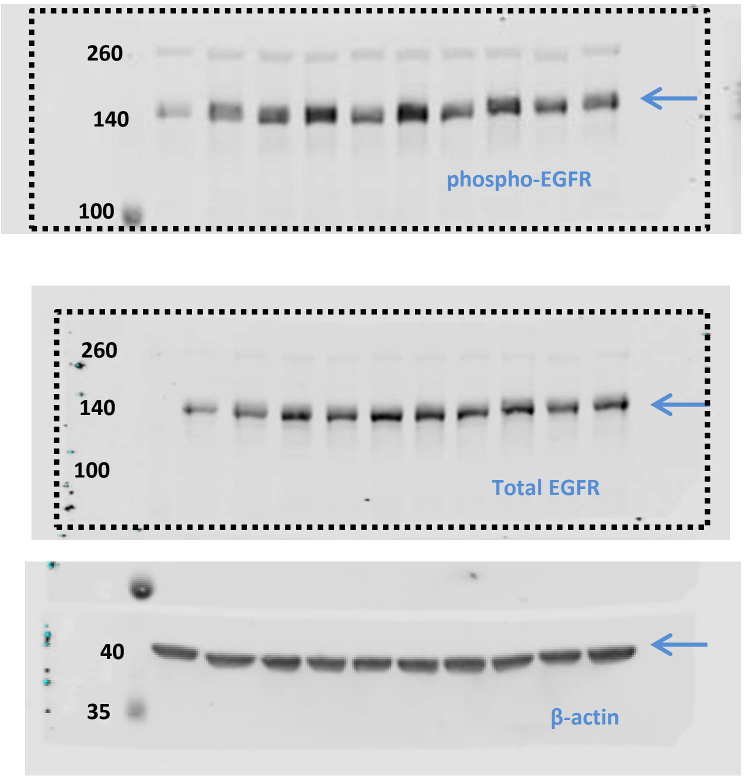

Supplementary Figure 7b: Uncropped version of blots presented in main Figure 1(c). The fresh membrane was cut below 100 kDa to blot for phospho-EGFR and beta-actin. The membrane was stripped and re-probed for total EGFR. Blue arrows indicate the location of the bands-of-interest. Dotted lines indicate membrane periphery.

Supplementary Figure S8: Figure 3a

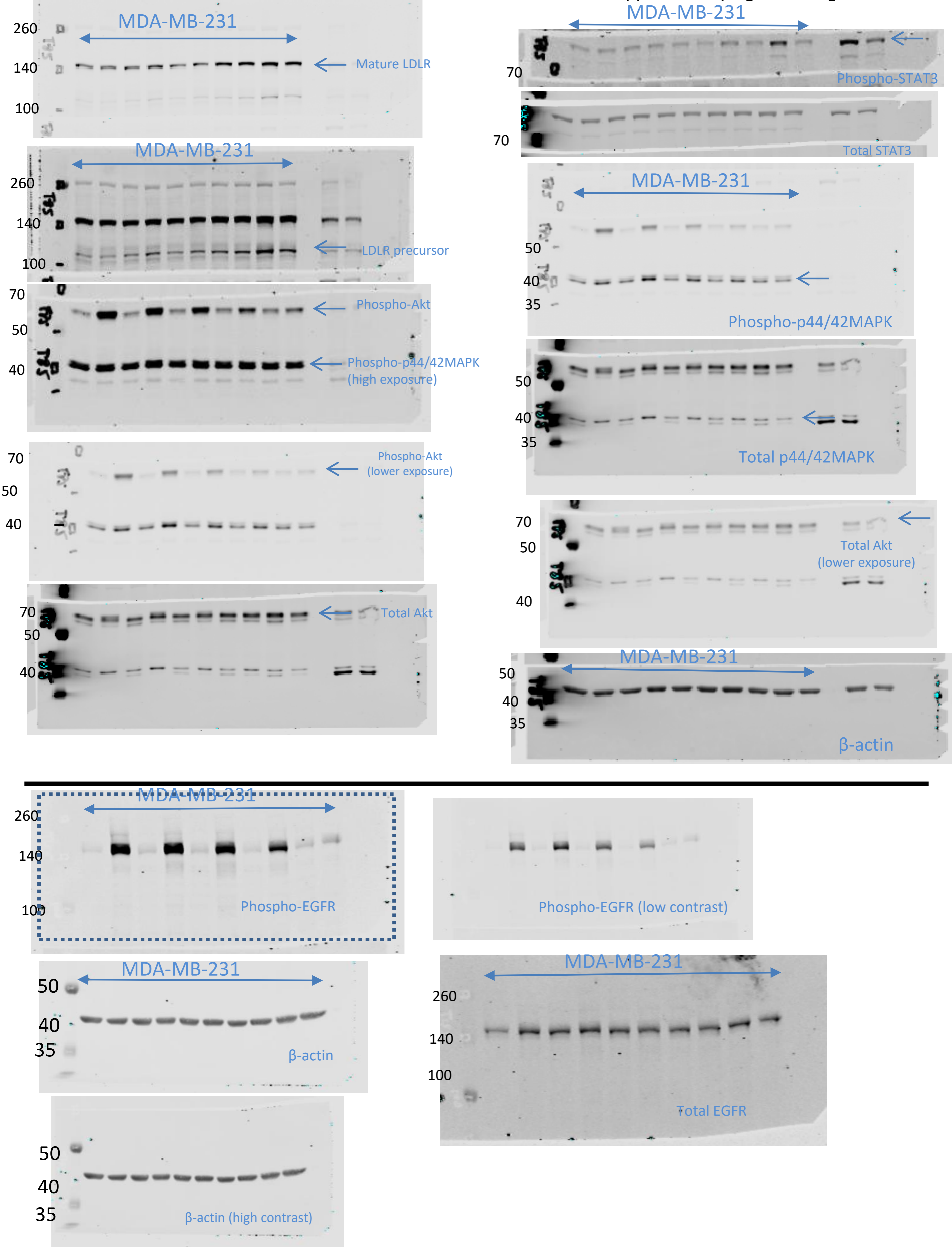

Supplementary Figure 8: Uncropped version of blots presented in main Figure 3(a). Top: The fresh membrane was cut below 100 kDa and below 70 kDa to blot for LDLR, STAT3, Akt and MAPK. Phospho-MAPK/Akt/STAT3 (rabbit source) and total MAPK/Akt/STAT3 (mouse source) probing were done concurrently using secondary antibodies conjugated to different fluorochromes. The LDLR (precursor) image was acquired at the same time as the LDLR (mature) image but exposed for longer. The membrane was stripped, cut and re-probed for β-actin. Bottom: The same samples were used to run a separate gel/blot for EGFR and β-actin. The blot was stripped and re-probed for total EGFR. The image for total EGFR was acquired as shown. Dotted lines indicate the edges of blot where they are not clearly visible. Blue arrows indicate the location of the bands-of-interest. Multiple exposures are shown for blots that were either taken with a higher contrast or that do not have backgrounds sufficiently dark to show the membrane periphery.

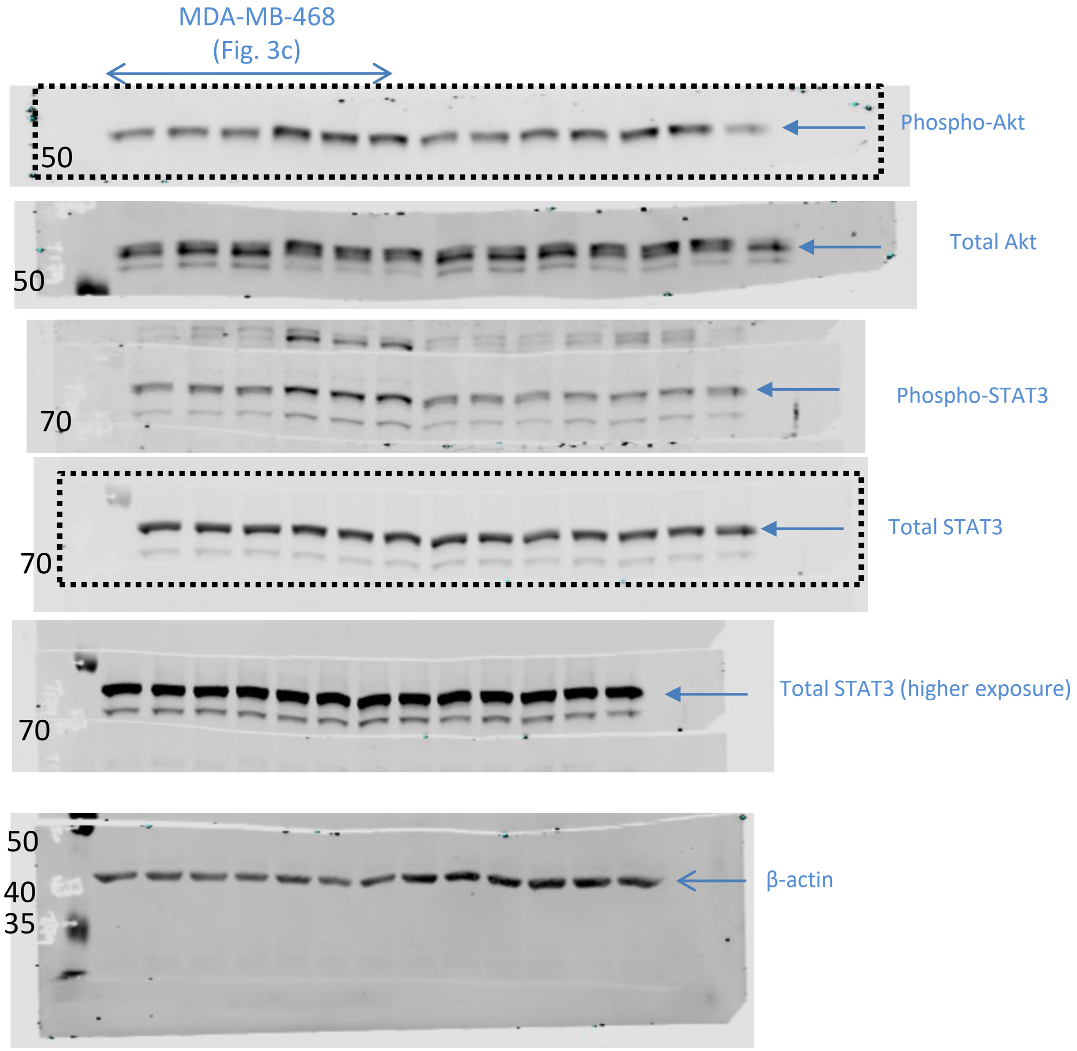

Supplementary Figure 9: Uncropped version of blots presented in main Figure 3(c). The fresh membrane was cut below 100 kDa, 70 kDa and 50 kDa to blot for STAT3, Akt and β-actin. Phospho-Akt/STAT3 (rabbit source) and total Akt/STAT3 (mouse source) probing were done concurrently using secondary antibodies conjugated to different fluorochromes. Blue arrows indicate the location of the bands-of-interest. Multiple exposures are shown for blots that were either taken with a higher contrast or that do not have backgrounds sufficiently dark to show the membrane periphery. Dotted lines indicate membrane periphery.

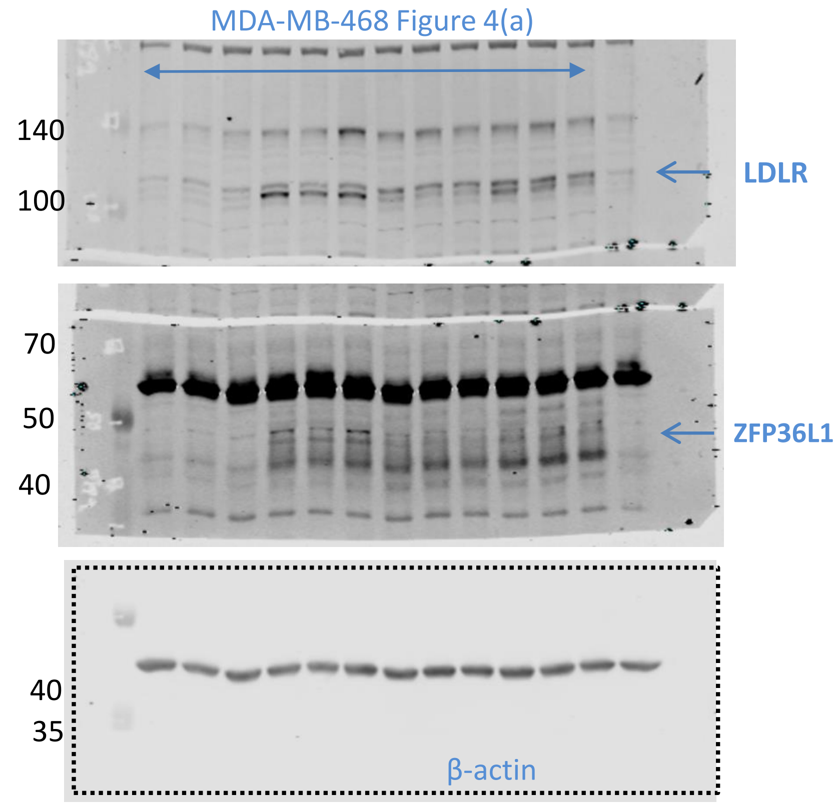

Supplementary Figure 10: Uncropped version of blots presented in main Figure 4(a). Two separate gels were run using the same samples. Top: The fresh membrane was cut below 100 kDa to blot for LDLR and ZFP36L1. The membrane was then stripped and re-probed for β-actin. Bottom: The fresh membrane was cut below 70 kDa to blot for STAT3 and MAPK. Phospho-MAPK/STAT3 (rabbit source) and total MAPK/STAT3 (mouse source) probing were done concurrently using secondary antibodies conjugated to different fluorochromes. The membrane was stripped, cut and re-probed for β-actin. Blue arrows indicate the location of the bands-of-interest. Dotted lines indicate membrane periphery.

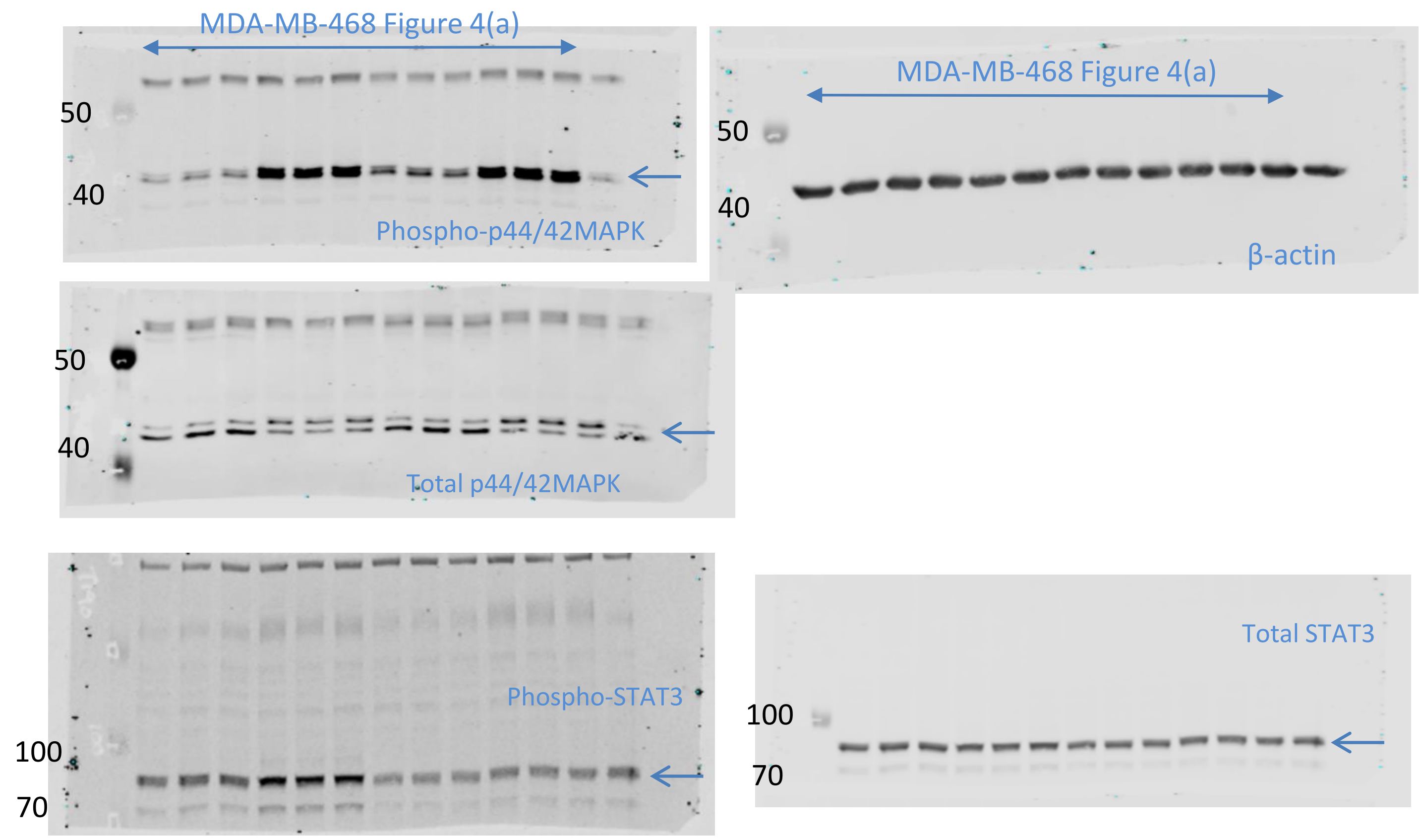

Supplementary Figure S11: Figure 4c

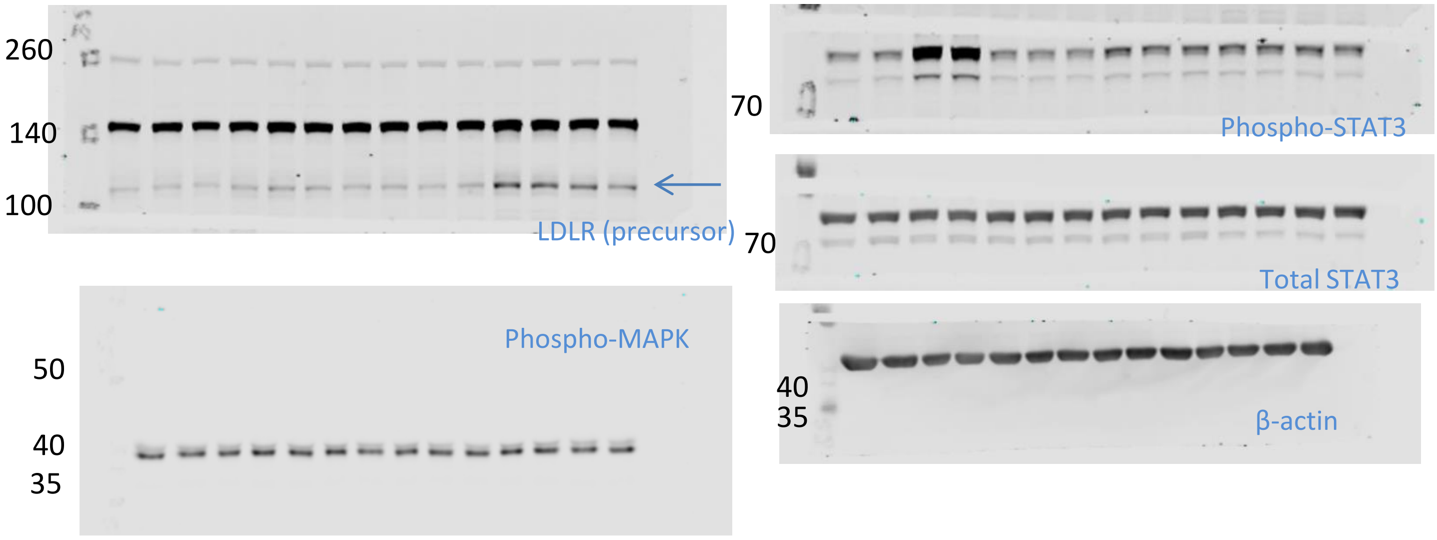

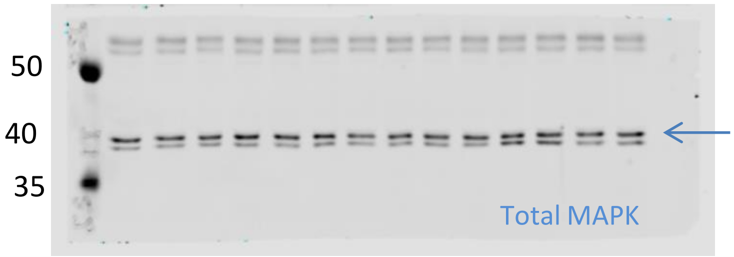

Supplementary Figure 11: Uncropped version of blots presented in main Figure 4(c). The fresh membrane was cut below 100 kDa and below 70 kDa to blot for LDLR, STAT3 and MAPK. Phospho-MAPK/STAT3 (rabbit source) and total MAPK/STAT3 (mouse source) probing were done concurrently using secondary antibodies conjugated to different fluorochromes. The membrane was stripped, cut and re-probed for β-actin. Blue arrows indicate the location of the bands-of-interest. Multiple exposures are shown for blots that were either taken with a higher contrast or that do not have backgrounds sufficiently dark to show the membrane periphery.

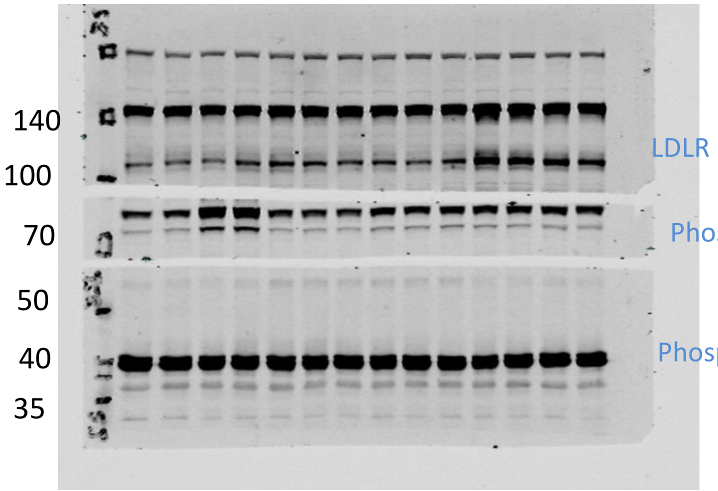

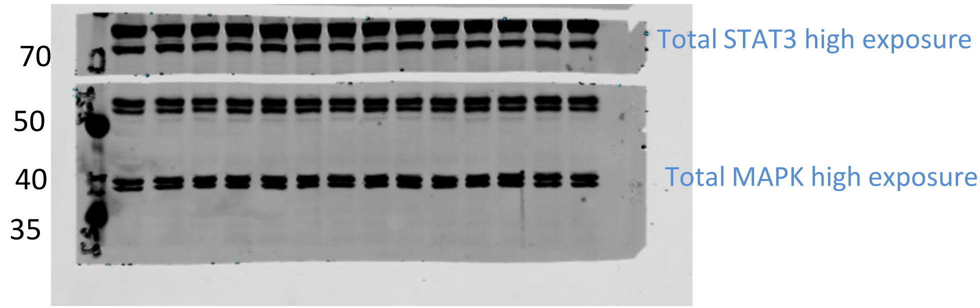

Supplementary Figure S12: Figure 5a

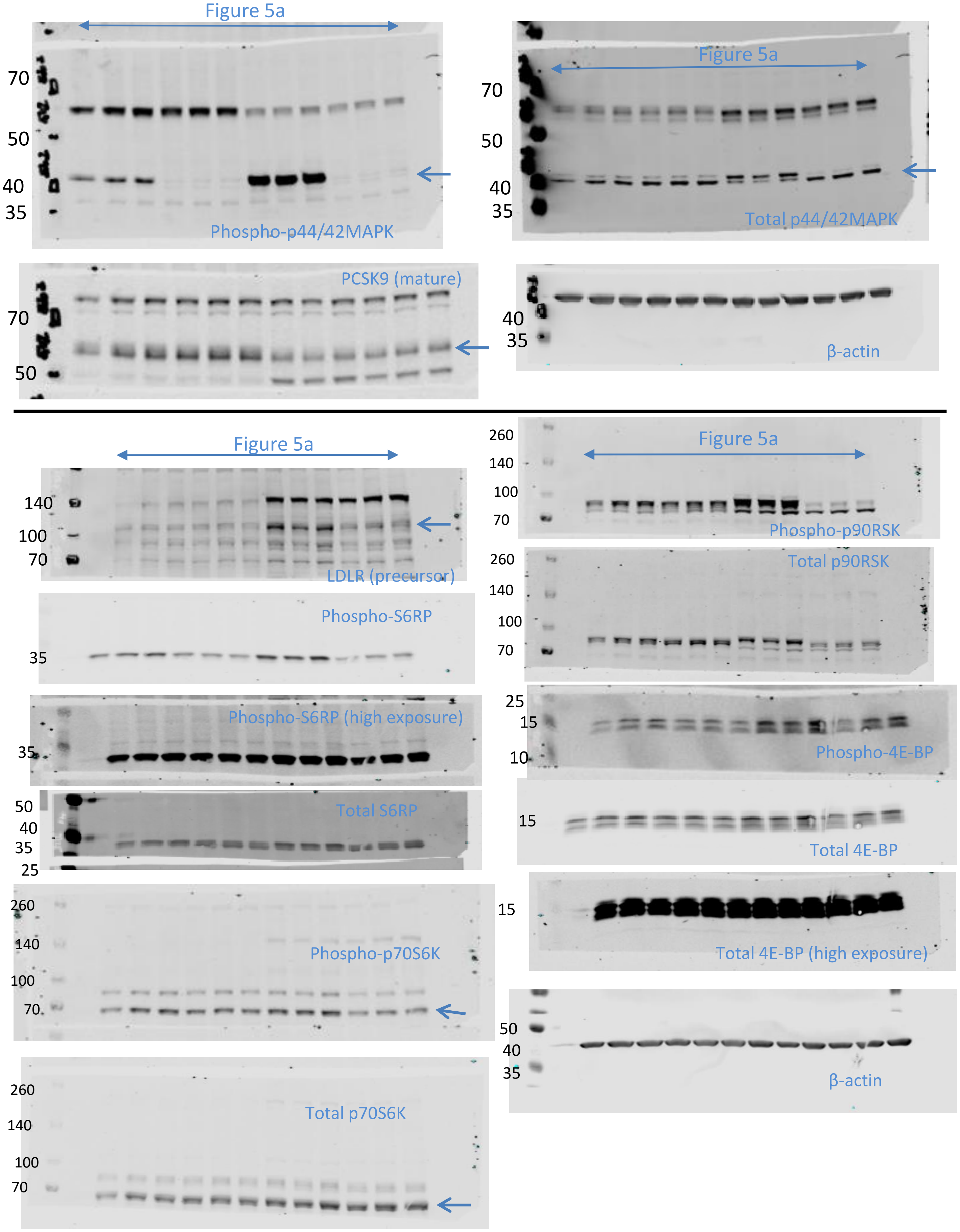

Supplementary Figure 12: Uncropped version of blots presented in main Figure 5(a). The same samples were run twice to make two separate gels/blots. Top half: The fresh membrane was cut below 70 kDa to blot for MAPK. Phospho-MAPK (rabbit source) and total MAPK (mouse source) probing were done concurrently using secondary antibodies conjugated to different fluorochromes. The membrane was stripped, cut and re-probed for PCSK9 and then, β-actin. Bottom half: The fresh membrane was cut below 70 kD, above 50 kDa, and at 25 kDa to blot for LDLR, S6RP and phospho-4E-BP. Phospho-S6RP (rabbit source) and total S6RP (mouse source) probing were done concurrently using secondary antibodies conjugated to different fluorochromes. The membrane were stripped and re-probed for in order; phospho-p90RSK and total 4E-BP, phospho-p70S6K and β-actin, total p90RSK and total p70S6K. Blue arrows indicate the location of the bands-of-interest. Multiple exposures are shown for blots that were either taken with a higher contrast or that do not have backgrounds sufficiently dark to show the membrane periphery.

Supplementary Figure S13: Figure 6a

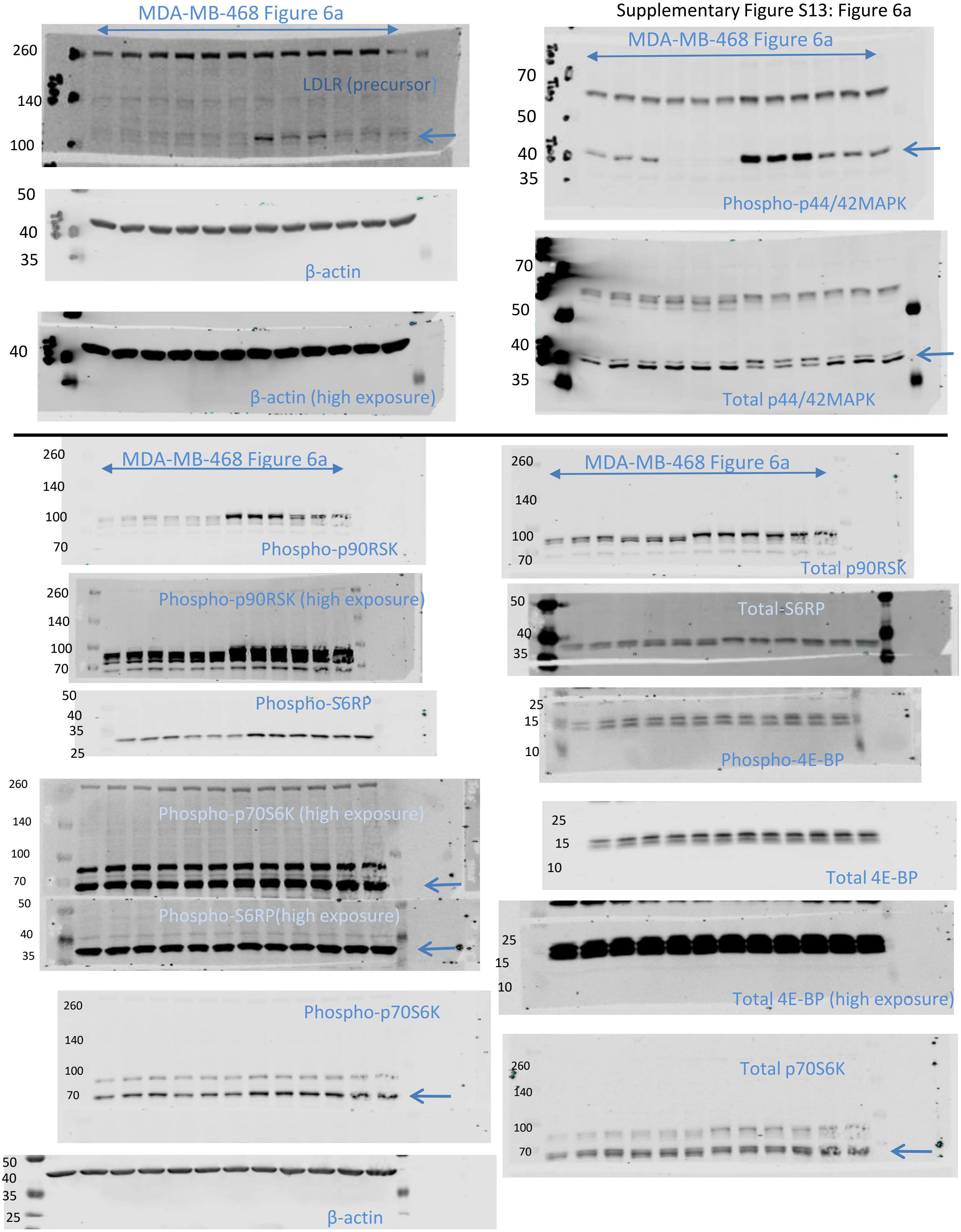

Supplementary Figure 13: Uncropped version of blots presented in main Figure 6(a). The same samples were run twice to make two separate gels/blots. Top half: The fresh membrane was cut below 100 kDa to blot for LDLR and MAPK. Phospho-MAPK (rabbit source) and total MAPK (mouse source) probing were done concurrently using secondary antibodies conjugated to different fluorochromes. The membrane was stripped and re-probed for β-actin. Bottom half: The fresh membrane was cut below 70 kD and above 25 kDa to blot for phospho-p90RSK, S6RP and phospho-4E-BP. Phospho-S6RP (rabbit source) and total S6RP (mouse source) probing were done concurrently using secondary antibodies conjugated to different fluorochromes. The membranes were stripped and re-probed for in order; phospho-p70S6K and total 4E-BP, total p90RSK and β-actin, and then total p70S6K. Blue arrows indicate the location of the bands-of-interest. Multiple exposures are shown for blots that were either taken with a higher contrast or that do not have backgrounds sufficiently dark to show the membrane periphery.

Supplementary Figure S14: Figure 6d

Supplementary Figure 14: Uncropped version of blots presented in main Figure 6(d). The same samples were used to run a separate gel/blot for phospho- and total p44/42MAPK. Top: Phospho-MAPK (rabbit source) and total MAPK (mouse source) probing were done concurrently using secondary antibodies conjugated to different fluorochromes. The membrane was stripped and re-probed for β-actin. Bottom: The fresh membrane was cut below 100 kDa to blot for LDLR and at 50 kDa to blot for ZFP36L1. The membrane was then stripped and re-probed for β-actin. Blue arrows indicate the location of the bands-of-interest. Multiple exposures are shown for blots that were either taken with a higher contrast or that do not have backgrounds sufficiently dark to show the membrane periphery.

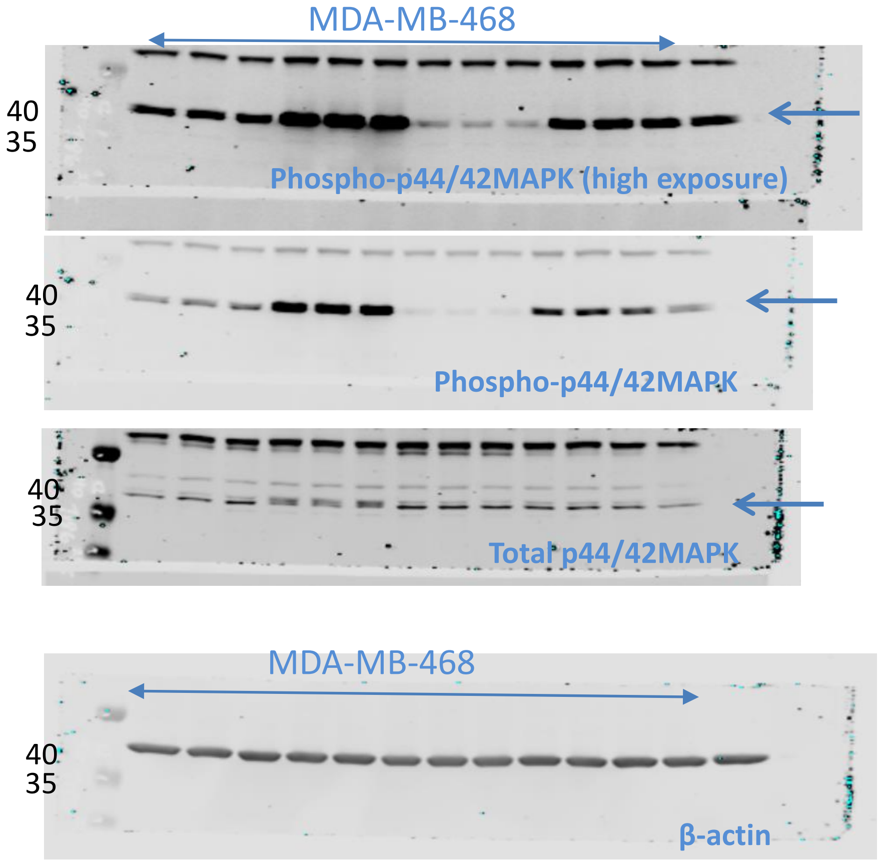

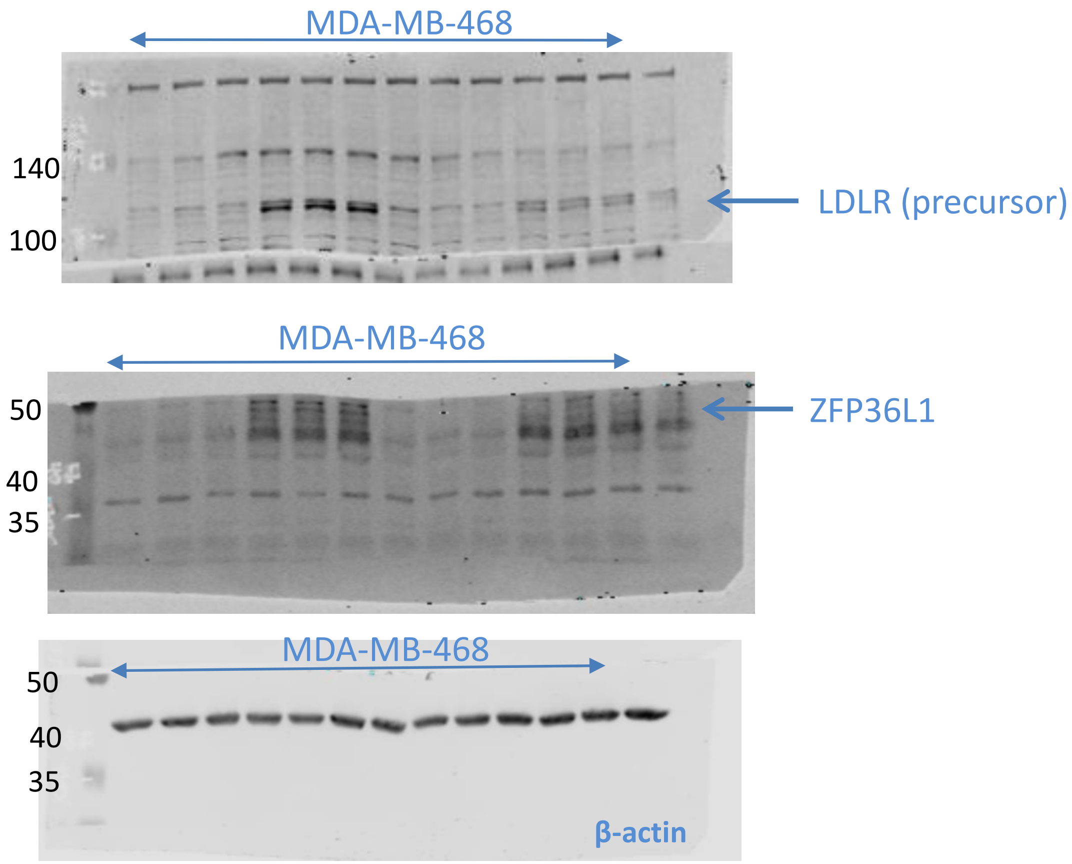

Supplementary Figure S15: Figure 7a

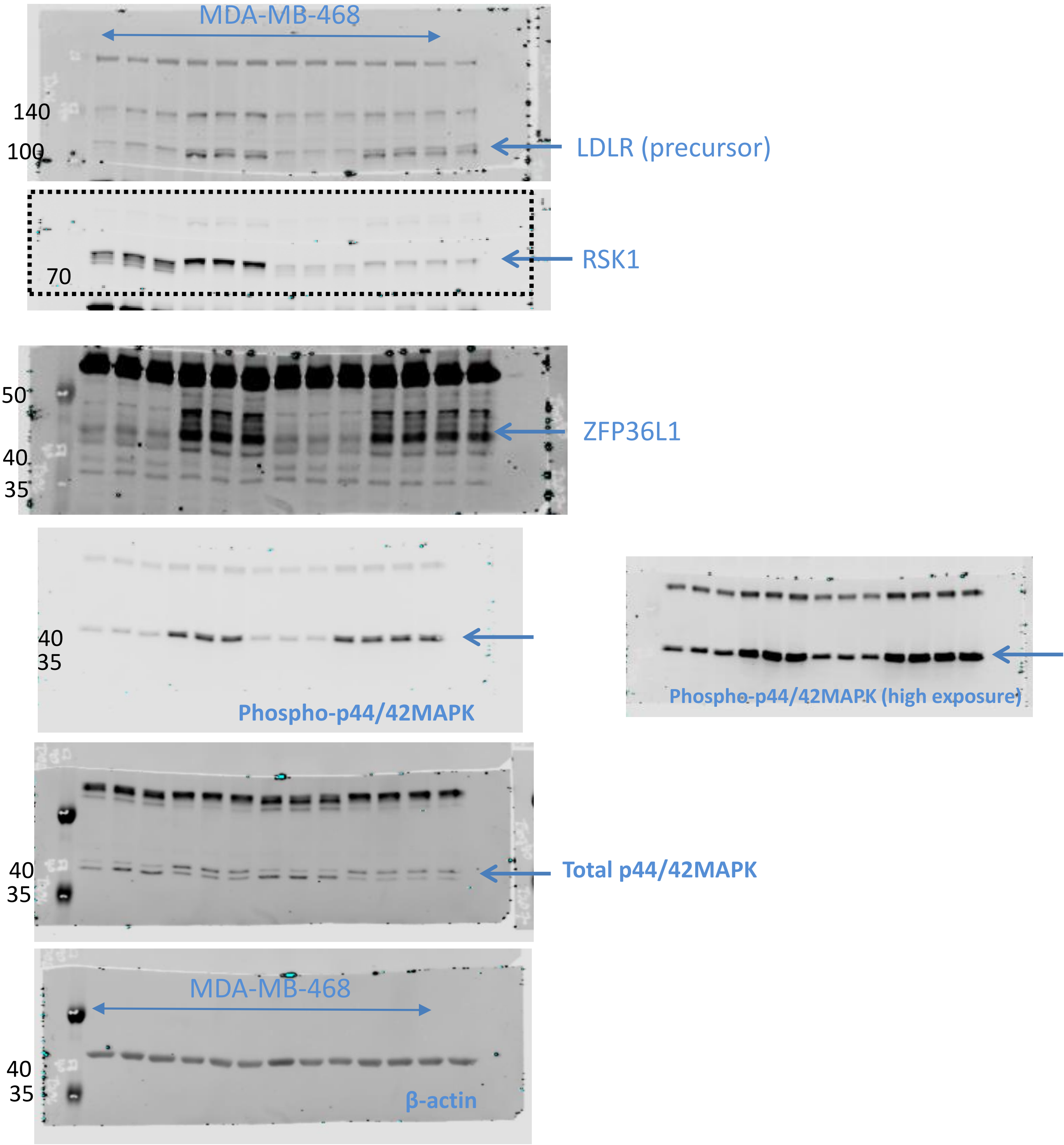

Supplementary Figure 15: Uncropped version of blots presented in main Figure 7(a). The fresh membrane was cut below 100 kDa to blot for LDLR, below 70 kDa to blot for RSK1 and at 50 kDa to blot for ZFP36L1. The membrane was then stripped and re-probed for phospho- and total p44/42 MAPK, followed by stripping and re-probing for β-actin. Phospho-MAPK (rabbit source) and total MAPK (mouse source) probing were done concurrently using secondary antibodies conjugated to different fluorochromes. Blue arrows indicate the location of the bands-of-interest. Dotted lines indicate membrane periphery. Multiple exposures are shown for blots that were either taken with a higher contrast or that do not have backgrounds sufficiently dark to show the membrane periphery.

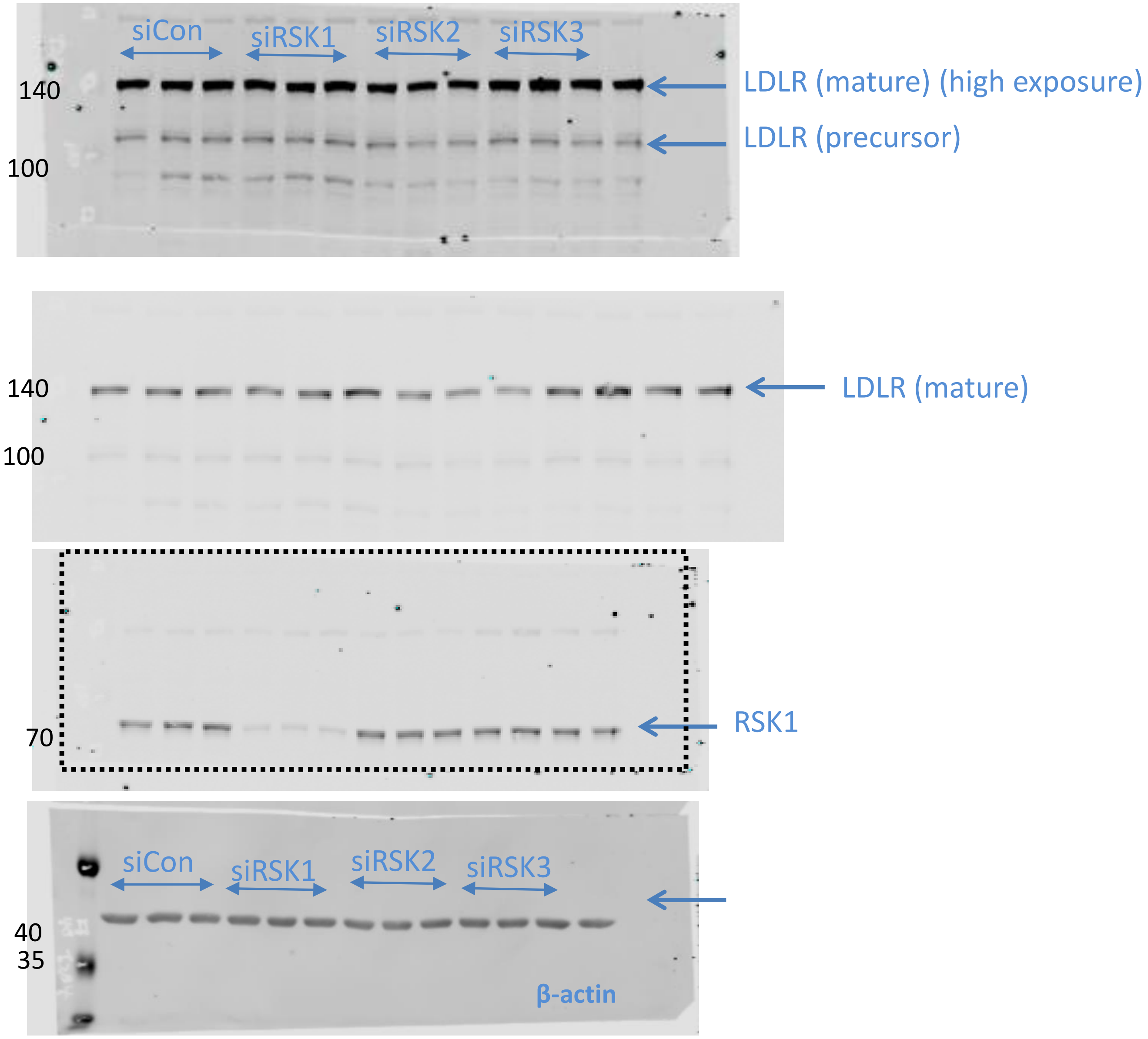

Supplementary Figure 16: Uncropped version of blots presented in main Figure 7(c). The fresh membrane was cut below 100 kDa to blot for LDLR, below 70 kDa to blot for RSK1 and at 50 kDa to blot for β-actin. Blue arrows indicate the location of the bands-of-interest. Dotted lines indicate membrane periphery.

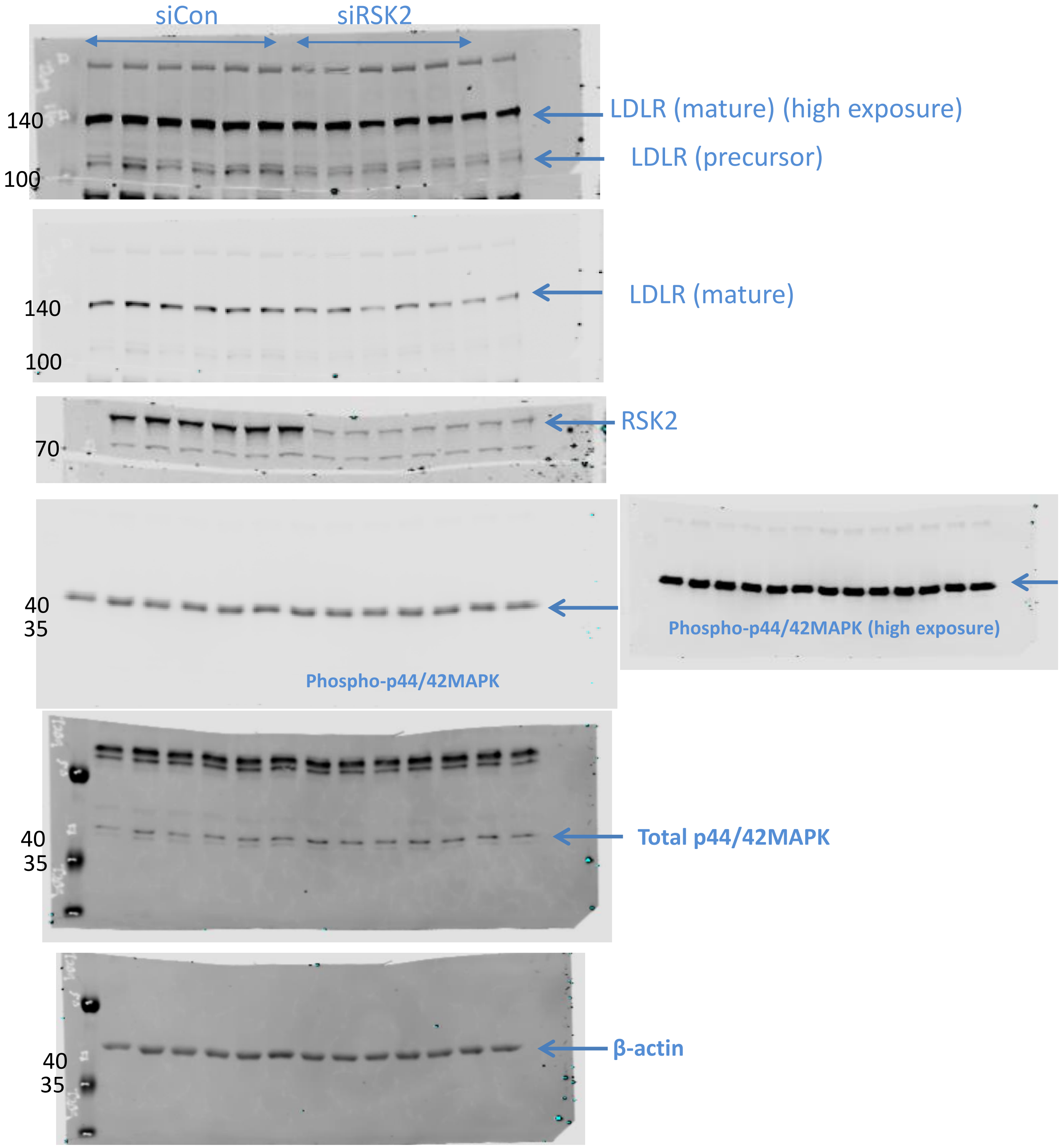

Supplementary Figure 17: Uncropped version of blots presented in main Figure 7(d). The fresh membrane was cut below 100 kDa to blot for LDLR, below 70 kDa to blot for RSK2 and at 50 kDa to blot for for phospho- and total p44/42 MAPK, followed by stripping and re-probing for β-actin. Phospho-MAPK (rabbit source) and total MAPK (mouse source) probing were done concurrently using secondary antibodies conjugated to different fluorochromes. Blue arrows indicate the location of the bands-of-interest. Multiple exposures are shown for blots that were either taken with a higher contrast or that do not have backgrounds sufficiently dark to show the membrane periphery.
